# Pyrrolic and Dipyrrolic Chlorophyll Degradation Products in Plants and Herbivores

**DOI:** 10.1002/chem.201905236

**Published:** 2020-04-28

**Authors:** Marcel Ritter, Vincensius S. P. Oetama, Daniel Schulze, Katrin Muetzlaff, Anja K. Meents, Raphael A. Seidel, Helmar Görls, Matthias Westerhausen, Wilhelm Boland, Georg Pohnert

**Affiliations:** ^1^ Friedrich Schiller University Jena Institute of Inorganic and Analytical Chemistry Lessingstr. 8 07743 Jena Germany; ^2^ Max Planck Institute for Chemical Ecology Hans-Knöll-Str. 8 07745 Jena Germany; ^3^ Friedrich Schiller University Jena Institute of Inorganic and Analytical Chemistry Humboldtstr. 8 07743 Jena Germany

**Keywords:** bilirubin oxidation end products, chlorophyll degradation, natural products, porphyrinoids, propentdyopents

## Abstract

The degradation of chlorophyll, the omnipresent green pigment, has been investigated intensively over the last 30 years resulting in many elucidated tetrapyrrolic degradation products. With a comparison to the degradation of the structurally similar heme, we hereby propose a novel additional chlorophyll degradation mechanism to mono‐ and dipyrrolic products. This is the first proof of the occurrence of a family of mono‐ and dipyrrols in leaves that are previously only known as heme degradation products. This product family is also found in spit and feces of herbivores with specific metabolomic patterns reflecting the origin of the samples. Based on chromatographic and mass spectrometric evidence as well as on mechanistic considerations we also suggest several tentative new degradation products. One of them, dihydro BOX A, was fully confirmed as a novel natural product by synthesis and comparison of its spectroscopic data.

## Graphical abstract

**On its degradation**: Evidence for a further pathway of chlorophyll degradation was found in plants and herbivores. Mono‐ and dipyrrolic compounds, known from heme degradation, were detected and quantified in leaves, spit and feces of herbivores. Novel natural products were detected and the structure of one, dihydro BOX A, was proven by synthesis.

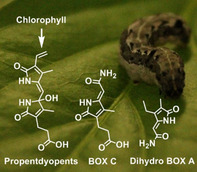

## Introduction

**Scheme 1:**
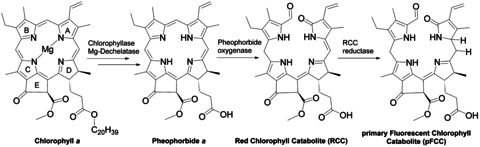
Enzymatic chlorophyll degradation in plants.

Chlorophyll (Chl) as a major pigment in plants, bacteria, and algae, is produced and degraded annually on a 10^12^ kilogram scale.^1^ The different Chl species exhibit a porphyrin structure with an additional 5‐ring and Mg^2+^ as center ion. Except for Chl *c*, one double bond in the d‐ring is hydrogenated, resulting in chlorins or dihydro porphyrins.^2^ The most abundant Chl *a* exhibits four methyl, one ethyl and one vinyl substituents, as well as a methyl and a phytol ester (Scheme 1).^3^, ^4^

If the light absorption process in photosynthetic plant tissue is overexcited, for example, under intensive light conditions, or the energy transfer is impaired, the absorbed energy can trigger the production of reactive oxygen species (ROS).^5^ Since these products are cell toxic, the metabolism of Chl is highly regulated in plants.^6^ In autumn, a well‐studied chlorophyll degradation becomes visible in a beautiful diversity of colors from senescent leaves.^7^, ^8^, ^9^, ^10^, ^11^ However, Chl degradation can also be induced upon herbivore attack or pathogen infestation.^12^, ^13^

In the first enzymatic degradation steps, the phytol ester is cleaved by chlorophyllase leading to chlorophyllide, followed by a demetallation by Mg‐dechelatase resulting in pheophorbide *a* (Scheme 1).^14^ As a key step, the macrocycle is opened between the A and B rings by pheophorbide oxidase leading to red chlorophyll catabolites (RCCs).^15^ A double bond reduction at the bridge between the A and D rings leads to primary fluorescent chlorophyll catabolites, which can be further modified by so far unknown enzymes or reactions to non‐fluorescent chlorophyll catabolites (NCCs, Scheme 1).^16^, ^17^ Even if the pathway and functions are not yet understood, structurally different NCCs were detected especially in the vacuoles of plants and are considered end products of the chlorophyll degradation process.^18^, ^19^, ^20^, ^21^, ^22^

The further fate of Chl degradation products during abiotic decay as well as herbivory remains open to a large extent. Previous studies showed chlorophyll catabolite products in feces of herbivores that are also known from enzymatic Chl degradation in plants, for example, chlorophyllide and pheophorbide.^23^, ^24^ However, RCCs or similar compounds with an open macrocycle were not found so far in such samples since anaerobic conditions in the gut hinder potential oxidative reactions to open the macrocycle. As soon as the catabolites are outside the digestive system, the condition is drastically changed to aerobic and the presence of light.^25^, ^26^ In this study, we address the hypothesis, that under such conditions in leaves and feces, a further Chl degradation to diverse mono‐ or dipyrrolic products occurs.

**Scheme 2:**
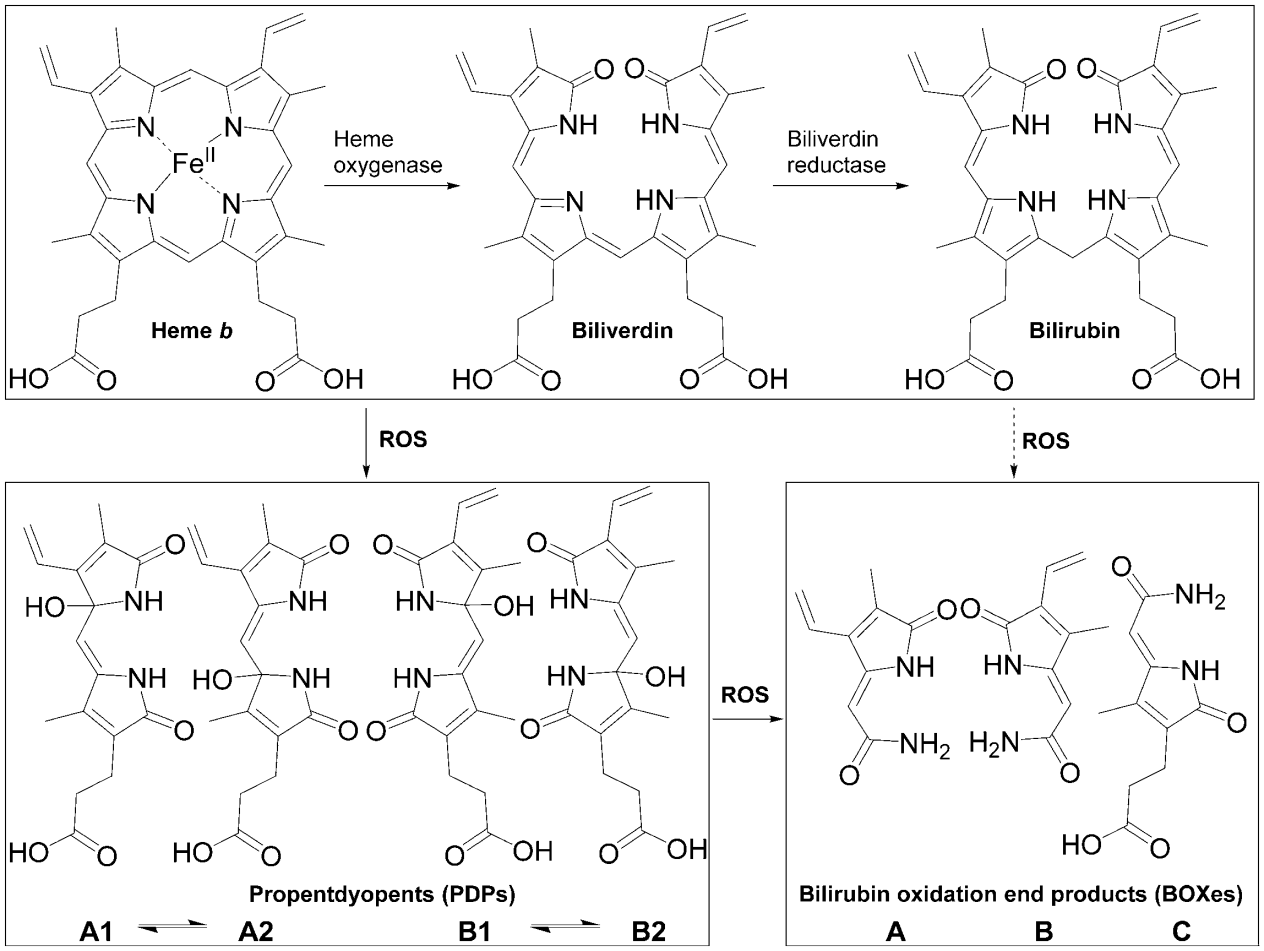
Enzymatic (top) and oxidative heme degradation by reactive oxygen species (ROS) to PDPs and BOXes.

A similar degradation is reported for a structurally related pigment of life, heme.^27^ The enzymatic degradation through biliverdin to bilirubin shows striking similarities to the early steps of Chl degradation (Scheme 2).^28^, ^29^ In mammals most of the bilirubin is conjugated and excreted into the bile,^30^ but an additional oxidative degradation occurs. This ROS‐mediated breakdown leads to dipyrrolic propentdyopents (PDPs)^31^ and monopyrrolic bilirubin oxidation end products (BOXes, Scheme 2).^32^, ^33^, ^34^ BOX A and B exhibit an intact vinyl pyrrole and differ only in the position of vinyl and methyl group, whereas BOX C derives from the propionic acid pyrrole. The dipyrrolic PDPs also differ in the position of their vinyl and methyl groups. In addition, two isomers in each structural group are observed (1 for OH at vinyl pyrrole and 2 for OH at propionic acid pyrrole). Both isomers of each group are in an equilibrium and are proven intermediates to BOXes.^31^ Those oxidative heme degradation products were quantified in nano‐ to micromolar concentrations with a 20–80 times higher abundance for PDPs compared to BOXes in bile, gallstones and the cerebrospinal fluid of stroke patients.^31^, ^35^, ^36^ Heme degradation products are involved in vessel constrictions as stroke complication and act as effectors in the liver.^35^, ^36^, ^37^

Since the building blocks, biosynthesis and enzymatic degradation of heme and chlorophyll show a high similarity, we searched for similar oxidative degradation pathways to pyrrols and dipyrrols for Chl.

We investigated chlorophyll degradation products in sweet potato leaves as well as in the spit and feces of the generalist herbivore *Spodoptera littoralis*. In vitro chlorophyll degradation was utilized to assign the structures. We could find PDPs and BOXes in the completely new context of Chl degradation and additionally detected new Chl catabolites, of which we proved the structure of one of them by synthesis.

## Results and Discussion

### In vitro chlorophyll *a* degradation

**Scheme 3:**
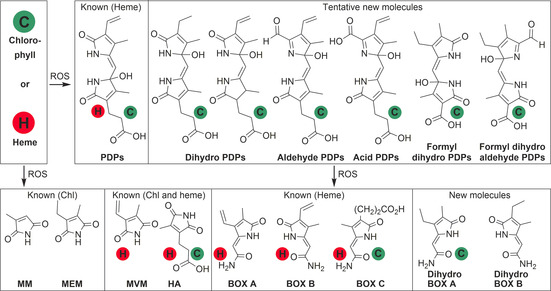
Overview of heme and chlorophyll degradation products, which are known from heme and chlorophyll (Chl) or introduced in this study. H in red circle marks the products found in heme degradation and C in green circle marks the products that we found in leaves and/or spit/feces of herbivores (ROS=reactive oxygen species, PDPs=propentdyopents, MM=methyl maleimide, MEM=methyl ethyl maleimide, MVM=methyl vinyl maleimide, HA=hematinic acid, BOX=bilirubin oxidation end product). For simplification, the four known PDPs (Scheme 2) were considered collectively and only one molecule is shown. Accordingly, the new proposed but not fully characterized PDPs represent the different isomers regarding vinyl/methyl and OH‐group position. The proposed structures are in accordance with considerations on the possible degradation mechanisms in analogy to heme degradation, high resolution MS data (Table S1) and retention time in RP‐HPLC of PDPs (Figure S13). Dihydro PDPs might arise from incorporation of the ethyl b‐ring as shown or alternatively by the saturated d‐ring of Chl. Since the macrocycle opening of Chl results in an aldehyde group (Scheme 1) instead of a carbonyl group in heme degradation (Scheme 2), we further propose PDPs with an aldehyde or acid group. As known from chlorophyllin, the additional five‐ring E of Chl can be oxidized and cleaved resulting in a formic acid group. This would lead to the formyl PDPs. For three of the proposed PDP species (dihydro, aldehyde and acid PDPs) the highest amounts were found in the leaves and not in spit or feces (data not shown).

To identify pyrrolic and dipyrrolic Chl degradation products, we initially performed a model oxidation in vitro. We used a procedure established for the investigation of heme degradation to address the lower molecular weight fraction of Chl degradation products.^31^, ^32^, ^39^ Chl *a*, suspended in sodium hydroxide solution, was neutralized with hydrogen chloride before oxidation with hydrogen peroxide. Since the reaction is rather unspecific, diverse products with mono‐ and dipyrrolic structures were found by UHPLC‐MS in traces after one day. Prolonged incubation over several days did not lead to a substantial increase of these products. Potential reasons for the low detected amounts are the insufficient solubility of Chl in water, the high oxidative potential needed to break the macrocycle and, once the macrocycle is broken, a comparably easier further reaction of degradation products. To avoid this, sodium hydroxide in methanol was used with 10 % hydrogen peroxide, which resulted in higher amounts of degradation products. With this optimized method, we could prove by UHPLC/HR‐MS and the addition of standards the occurrence of PDP B1/B2^31^ as well as of BOX A, B, C^34^, ^40^, ^41^ and hematinic acid (HA)^34^ as degradation products of Chl *a* in vitro (Scheme 3, see Supporting Information, Figures S7–S12). The finding of BOX A seems surprising, considering that the corresponding part of Chl holds an ethyl instead of a vinyl group. To result in BOX A, the initial macrocycle opening has to occur at a different pyrrole bridge and not between rings A and B as in the enzymatic ring opening or the oxidative pathway known for heme degradation.

We tentatively identified dihydro BOX A and B based on their mass spectra and chromatographic retention times (Supporting information, Figures S10). We also detected products that were in accordance with dipyrrolic structures, similar to PDPs (Supporting information, Figure S13, Table S1).

### Confirmation of proposed structures

To confirm the structure of the proposed molecules, the degradation products needed to be isolated in higher amounts. Given the low yield, the degradation of chlorophyll *a* was not feasible and chlorophyllin was used instead. Due to the better water solubility of chlorophyllin, the oxidation was carried out in water without sodium hydroxide. Since chlorophyllin is a mixture of different chlorins and porphyrins,^38^, ^42^ an even higher variety in products was observed, which overall only led to a small increase in the amount of degradation products. After oxidation, a chloroform extraction was performed to obtain a BOXes‐rich extract. The following preparative HPLC‐UV yielded fractions of the proposed molecules, dihydro BOX A and B. With NMR and MS‐MS experiments, the general BOX structure was proven, but due to overlaying signals of impurities, a synthetic approach was used to confirm the complete structure and configuration.

**Scheme 4:**
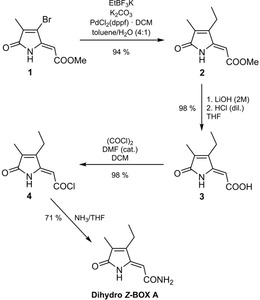
Synthesis of dihydro *Z*‐BOX A through a four‐step procedure (THF = tetrahydrofurane, DCM = dichloromethane, DMF = *N*,*N*‐dimethylformamide, dppf = 1,1'‐bis(diphenylphosphino)ferrocene).

Following the published synthesis of BOX A, dihydro BOX A was synthesized and fully characterized.^40^, ^41^ Starting from methyl‐(*Z*)‐2‐(3‐bromo‐4‐methyl‐5‐oxo‐1,5‐di‐hydro‐2*H*‐pyrrol‐2‐ylidene)ethanoate (**1**) the synthesis of dihydro BOX A succeeds through four synthetic steps with a total yield of 64 % (Scheme 4). The introduction of an ethyl group was performed with a Suzuki–Miyaura cross‐coupling reaction and resulted in high yield utilizing a palladium complex with sterically demanding 1,1'‐bis(diphenyl‐phosphino)ferrocene (dppf) as catalyst.^43^ Further conversion of coupling product **2** by cleavage of the methyl ester, transformation to the corresponding acyl chloride **4** and subsequent formation of the amide functionality by reaction with ammonia provided analytically pure dihydro *Z*‐BOX A.

**Figure 1:**
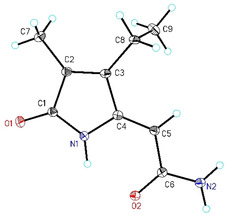
Molecular structure and numbering scheme of dihydro BOX A. The ellipsoids represent a probability of 30 %, H atoms are shown with arbitrary radii.

Structure and *Z*‐configuration were confirmed by single‐crystal X‐ray diffraction analysis. Structural parameters of the molecular structure shown in Figure 1 are similar to those published for *Z*‐BOX A with the exception of bond length and angle of the ethyl group. With a bond length of 152.2(4) pm the C8−C9 bond exhibits a typical single bond length and the C3‐C8‐C9 bond angle of 112.4(2)° is slightly compressed in comparison to the vinyl group bond angle of 124.7(2)° reported for *Z*‐BOX A (see also Supporting Information). Similar to other BOXes, aggregation by formation of intermolecular N−H⋅⋅⋅O hydrogen bridges is shown in the crystalline state, underlining the ability of those compounds for strong non‐covalent interactions. With the addition of the synthetic standard to the in vitro chlorophyll *a* degradation the structure of the degradation product could be proven (Supporting Information, Figure S10). This finding also supports the potential existence of dihydro BOX B and dihydro PDPs.

### Products of oxidative chlorophyll degradation in leaves

**Figure 2:**
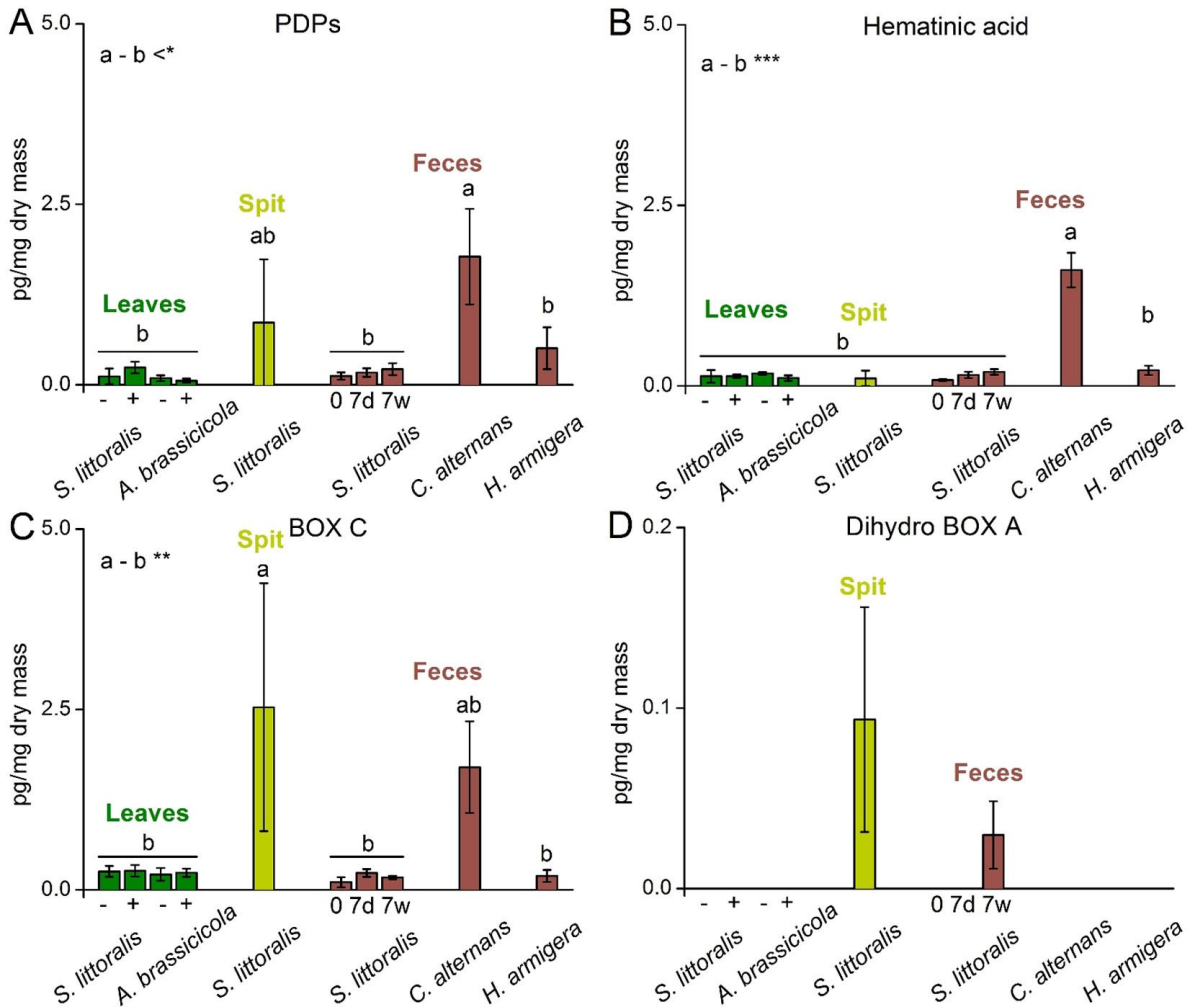
Comparison of different proposed chlorophyll degradation products in leaves (green), spit (yellow) and feces (brown). Leaves without induction are labelled with „−“, those induced with *S. littorals* or *A. brassicicola* are labelled with „+“. Feces were analyzed directly after collection (0 days) and after seven days in dry (7d) and wet conditions (7w). Note the different scales of the *y*‐axis in D. One‐way‐Anova with Bonferroni post‐hoc test was carried out for statistical analysis. Letters indicate statistical difference between the indicated groups a and b (* *p*=0.05, ** P=0.05, *** P=0.005).

To search for oxidative degradation products of Chl in vivo, sweet potato leaves (*Ipomoea batatas*) were extracted and analyzed by UHPLC‐MS. The analysis revealed the occurrence of Chl degradation products with identical chromatographic and mass spectrometric properties compared to those described above. Several structures are shared with heme degradation products. We unambiguously identified PDPs B1, and B2 as well as BOX C by comparison with authentic standards (Scheme 3).^31^, ^34^ These metabolites were quantified in amounts in the pg mg^−1^ dry mass range (Figure 2). In comparison, the Chl content in the sweet potato leaves was reported in low μg mg^−1^ dry mass.^44^ Similar to the observations made during the Chl *a* in vitro degradation, once the macrocycle is opened, the reactivity for further oxidative transformations is higher, potentially resulting in faster further reactions. Additionally, low molecular weight degradation products might be taken up or metabolized by the organism. We also detected LC‐MS‐evidence for unidentified dihydro BOXes as well as novel PDPs that were initially found in the in vitro degradation. In total, mass spectra HRMS and retention times pointed towards five new PDPs, and two new BOXes (Supporting Information Figure S13, Table S1).

PDPs occurred overall in 0.1 to 1 pg mg^−1^ dry mass, whereas BOX A and B could only be observed in traces. This is in accordance with the overall lower concentration of those products in heme degradation. There, PDPs as intermediates usually occurred in 20–80 times higher amounts than BOXes.^31^, ^36^ In contrast, BOX C occurred in higher amounts compared to PDPs in the leaves. This discrepancy points towards the existence of so far unknown PDP species as degradation intermediates. Peaks with matching masses of the five proposed PDPs that could act as such intermediates (Supporting Figure S13) were found in the investigated samples. Due to the lack of standards, only the peak areas obtained from LC‐MS can be compared, but the overall amounts of the proposed molecules can be estimated in the ng to high pg mg^−1^ dry mass range. In principle, it cannot be excluded that plant‐derived heme or bilins contribute to the observed break down products as well. Since the relative amounts of the detected products are similar to those observed in the in vitro Chl oxidation experiments we conclude that heme can, if at all, only contribute to a minor extend to the detected catabolic products. Further, the amount of clearly Chl derived products (green dots in Scheme 3) compared to those that could be derived from Chl as well as heme (green and red dots in Scheme 3) are similar, pointing also to a substantial or exclusive Chl‐origin.

According to literature, maleimides and hematinic acid were found in senescent barley and radish leaves, as well as lake sediments, which are considered first hints on low molecular weight oxidative degradation products.^44^, ^45^, ^46^, ^47^ In our study, HA was found in similar quantities as PDPs whereas maleimides could not be detected.

### Induction

The potential induction of Chl degradation products in response to herbivores and pathogens was investigated in detail in sweet potato leaves. To mimic natural stress conditions, leaves were either infested with the generalist herbivore *Spodoptera littoralis* or infected with the pathogenic fungus *Alternaria brassicicola*. After 30 min of herbivore feeding or 48 h of fungal inoculation, the leaves were harvested, dried and extracted. We observed no significant up‐regulation of Chl catabolites compared to the untreated leaves (Figure 2). Previous studies reported decreased Chl concentration after herbivore attack and infection with *A. brassicicola*.^13^ Our study does, however, not show an increased concentration of the defined oxidative degradation products indicating alternative degradation pathways being induced.

### Spit

To verify if the identified chlorophyll degradation products are found in the spit of insects and if their formation is continued during digestion, we investigated a well‐established plant/herbivore combination. Spit and feces of the tobacco horn worm *Spodoptera littoralis* that was feeding on sweet potato leaves were extracted and investigated with UHPLC‐HRMS. The content of Chl degradation products was then compared to that in the intact leaves.

Herbivore spit contained significantly higher amounts of PDPs and BOX C compared to leaves (Figure 2). This fits to the overall idea of a stepwise degradation, since those products can only occur after at least one breakage of a five‐membered ring. This could occur already in the leaves or in the spit of herbivores. A small amount of dihydro BOX A was also found in the spit. This compound was not detected in leaves and just in the feces after 7 days (Figure 2 D). In accordance, also concentrations of BOX A and B were low in the spit samples. Dihydro BOX B was not observed at all, which fits to the overall proposed pathway by known PDPs from which dihydro BOX B cannot be generated.

### Feces

Feces samples of *Spodoptera littoralis* were analyzed to monitor the production of Chl catabolites during and after plant digestion in the insect. Since it is known from literature, that a gut protein directly scavenges an early metabolite of Chl,^23^, ^24^, ^48^ the hypothesis was, that a substantial degradation is occurring by microbes after excretion. Therefore, samples were not only analyzed directly after excretion, but also incubated for one week at dry and wet conditions. In the initial samples, the overall amounts were rather low compared to leaves and spit. After one week, trends of increased amounts can be observed (Figure 2). Nevertheless, since the differences are not significant, further studies need to be carried out to investigate this into more detail.

To study whether those findings are specific for *S. littoralis*, the feces of two other herbivores (*Helicoverpa armigera* and *Chelymorpha alternans*) were also extracted and analyzed. Since both species were also reared on sweet potatoes, the amounts of degradation products found can be compared. The feces of *H. armigera* showed no significant differences to *S. littoralis* whereas *C. alterans* had overall higher amounts of degradation products indicating a certain species specificity (Figure 2).

## Conclusions

We present so far unknown chlorophyll degradation products in plants and herbivores. Mono‐ and dipyrrolic compounds, known from heme degradation, were for the first time connected to chlorophyll degradation and quantified in biological samples. They occur in the range of pg mg^−1^ dry weight with a species‐specific distribution that suggests enzymatic involvement in their formation. A series of novel molecules was proposed as extension to the existing degradation products and tentatively found in the investigated samples. The structure of dihydro BOX A, a so far unreported natural product, was proven by synthesis. Overall, this study opens a new field of research on the degradation of chlorophyll to smaller molecules.

## Experimental Section

**Materials and methods**

**Chemicals**: All solvents and compounds were purchased and used without further purification: Chlorophyll *a* (purified from *Anacystis nidulans*) and chlorophyllin (commercial grade) from Sigma–Aldrich (Munich, Ger), NaOH from Carl Roth (Karlsruhe, Ger), 30 % HCl from Merck (Darmstadt, Ger) and 50 % H_2_O_2_ from VWR (Darmstadt, Ger). HPLC gradient grade acetonitrile was obtained from VWR and water was purified with TKA microPure (Thermo Electron. Niederelbert, Ger). ULC gradient grade water and acetonitrile was purchased from Fisher Scientific (UK) and ULC formic acid was obtained from Biosolve B.V. (Valkenswaard, NL). For synthesis of dihydro BOX A all chemicals were used as purchased. Methyl (*Z*)‐2‐(3‐bromo‐4‐methyl‐5‐oxo‐1,5‐dihydro‐2*H*‐pyrrol‐2‐lidene) ethanoate (**1)** was prepared according to a literature procedure.^40^ Solvents were dried by standard methods if necessary. Tetrahydrofuran (THF) and toluene were distilled under nitrogen from sodium/benzophenone. Dichloromethane (DCM) was distilled under nitrogen from calcium hydride. *N*,*N*‐Dimethylformamide (DMF) was dried over calcium hydride and after filtration distilled under nitrogen. Deionized water was degassed by nitrogen sparging at reflux temperature. Merck silica gel 60 (0.040–0.063 mm) was used for column chromatography.

**Instrumentation**: To remove solvents and to dry samples, an evacuated centrifuge (Christ SpeedVac RVC 2–25) at 40 °C and a freeze dryer (Christ Alpha 1–2 LD) were used.

NMR data (^1^H; ^13^C) was collected on a 400 MHz Bruker Avance I or Avance III spectrometer using the residual solvent resonance of the solvents [D_1_]CDCl_3_ (^1^H *δ*=7.26; ^13^C *δ*=77.16), [D_6_]DMSO (^1^H *δ*=2.50; ^13^C *δ*=39.52) or [D_8_]THF (^1^H *δ*=1.72, 3.57; ^13^C *δ*=25.31, 67.39) as internal standard for referencing. Chemical shifts (δ) are reported in parts per million (ppm). A Bruker ALPHA Platinum‐ATR spectrometer was used to record IR spectra, intensities are reported as strong (s), medium (m), weak (w) or broad (br). Mass spectra were recorded on a Finnigan MAT SSQ 710 or ThermoFinnigan MAT 95 XL. Elemental analyses were performed using a Leco CHNS‐932 Elemental Analyzer.

For quantification and reaction monitoring a Dionex UltiMate 3000 UHPLC (Thermo Fisher Scientific, Leicestershire, UK) equipped with an Acquity UPLC BEH C18 column (1.7 μm, 100×2.1 mm) was used. The UHPLC was coupled to a Q‐Exactive Plus Orbitrap mass spectrometer (Thermo Fisher Scientific, Leicestershire, UK) and ionization was carried out with electrospray in positive ion mode. Solvent A contained 2 % acetonitrile in water with 0.1 % formic acid and solvent B 100 % acetonitrile with 0.1 % formic acid. The following gradient (time, vol% solvent B) was used: 0.0 min, 0 %; 0.5, 0 %; 1.0 min, 18 %; 8.0 min, 18 %; 9.0 min, 100 %; 10.9 min, 100 %; 11.0 min, 0 %; 13.0 min, 0 % with a flow rate of 0.4 mL min^−1^. External calibration using standards was performed for quantification.

For preparative separation a HPLC (Shimadzu LC‐8A, Kyoto, Jap) with a HTEC C18‐column (5 μm, 250 *x* 16 mm, Macherey–Nagel, Dueren, Ger) equipped with a SPD‐10AV UV–Vis detector measuring at 220 nm was used. Solvent A contained 2 % acetonitrile in water and solvent B 100 % acetonitrile. The gradients (time, vol % solvent B) were as follows: 0 min, 16 %; 50 min, 16 %, 51 min, 100 %, 56 min, 100 %; 58 min, 16 %; 66 min, 16 % with a flow rate of 6 mL min^−1^.

**Oxidative degradation**: The oxidative degradation of chlorophyll *a* was carried out based on published protocols for bilirubin.^32^, ^39^ To optimize the process, small test reactions of chlorophyll *a* were carried out in 5 m NaOH/H_2_O, in EtOH and in 2 m NaOH/MeOH with different amounts of H_2_O_2_ (1–10 %) and monitored by UHPLC‐HRMS. In the final procedure, chlorophyll *a* (3.45 mg, 3.86 μmol) was suspended in 2.5 mL of 2 m NaOH/MeOH and stirred for 24 h. The pH was adjusted to 7.5 with HCl before H_2_O_2_ was added to a final concentration of 10 % and stirred for 48 h. After extraction with chloroform to isolate a BOXes rich fraction, the water phase was subjected to solid phase extraction (30 mg Oasis hydrophilic lipophilic balanced cartridges; Waters, Manchester, United Kingdom). After elution with water and 20 % ACN/water, the fractions were dried under an N_2_‐flow and analyzed with UHPLC‐HRMS. Standards (PDPs, BOXes) were added to the extract to proof the occurrence of the compounds.

For preparative scale preparation of degradation products, the degradation of chlorophyllin (1 g, 1.38 mmol) was carried out in water (0.7 L) at pH 7.5 with an overall concentration of 1 % H_2_O_2_ for 1 d. Analogous to chlorophyll, a chloroform extraction followed by a solid phase extraction (6 g cartridge) were carried out. The fractions were dried with an evacuated centrifuge and preparative HPLC‐UV–Vis was carried out to isolate the product.

Purification of standards: PDPs, BOX C and hematinic acid were purified from bilirubin oxidation according to published protocols.^31^, ^34^ BOX A and B were synthesized as published.^40^, ^41^

**Synthesis and analytics of dihydro BOX A**

*Methyl (Z)‐2‐(3‐ethyl‐4‐methyl‐5‐oxo‐1,5‐dihydro‐2H‐pyrrol‐2‐ylidene) ethanoate (**2**)*: A solution of methyl‐(*Z*)‐2‐(3‐bromo‐4‐methyl‐5‐oxo‐1,5‐di‐hydro‐2*H*‐pyrrol‐2‐ylidene)ethanoate (**1**) (500 mg, 2.03 mmol), potassium ethyltrifluoroborate (332 mg, 2.44 mmol), [1,1’‐Bis(diphenylphosphino)‐ferrocene]palladium(II) dichloride complex with dichloromethane (83 mg, 0.10 mmol) and K_2_CO_3_ (842 mg, 6.10 mmol) in degassed toluene/water (25 mL, 4:1 v/v) was heated to 85 °C for 20 h in a nitrogen atmosphere. After cooling to room temperature, ethyl acetate (50 mL) was added and the solution was washed with water (2×20 mL) and brine (20 mL). The combined aqueous layers were extracted once with ethyl acetate (20 mL), the organic extracts then combined and dried over Na_2_SO_4_. The solvent was removed and the crude product purified by column chromatography (silica gel, ethyl acetate/*n*‐heptane, 1:1 v/v) to provide **2** as a pale yellow solid (374 mg, 94 %).

^**1**^ **H NMR** (400 MHz, CDCl_3_, 297 K): *δ*=8.99 (s, 1 H, N*H*), 5.37 (s, 1 H, C*H*), 3.77 (s, 3 H, COOC*H*_3_), 2.41 (q, *J=*7.6 Hz, 2 H, C*H*_2_CH_3_), 1.92 (s, 3 H, C*H*_3_), 1.13 (t, *J=*7.6 Hz, 3 H, CH_2_C*H*_3_) ppm; ^**13**^ **C{^1^H} NMR** (101 MHz, CDCl_3_, 297 K): *δ*=171.9, 167.9, 150.6, 146.3, 131.9, 93.1, 51.8, 17.8, 14.1, 8.6 ppm; **IR (ATR)**: ν=3251 (m; N−H), 1728 (m; C=O), 1680 (s; C=O, Amide I), 1637 (s; C=C) cm^−1^; **MS (DEI)**: *m*/*z* (%)=195 (100) [M]^+^, 163 (84), 148 (20), 135 (23), 108 (18); **Elemental analysis** (%): calcd for C_10_H_13_NO_3_ (195.22): C 61.53, H 6.71, N 7.18; found: C 61.47, H 6.71, N 7.11.

*(Z)‐2‐(3‐ethyl‐4‐methyl‐5‐oxo‐1,5‐dihydro‐2H‐pyrrol‐2‐ylidene)ethanoic acid (**3**)*: To a solution of **2** (300 mg, 1.54 mmol) in THF (6 mL) was added an aqueous solution of LiOH (2 m, 3 equiv) at 0 °C. After stirring at room temperature for 27 h, diluted hydrochloric acid was added at 0 °C till a pH value of 2 was reached. Ethyl acetate (20 mL) was added, the organic layer separated, and the aqueous layer extracted with ethyl acetate (3×10 mL). The combined organic layers were washed with brine, dried over Na_2_SO_4_ and the solvent removed in vacuo to provide **3** as a colorless solid (273 mg, 98 %).

^**1**^ **H NMR** (400 MHz, [D_6_]DMSO, 297 K): *δ*=12.57 (s, 1 H, COO*H*), 9.50 (s, 1 H, N*H*), 5.42 (s, 1 H, C*H*), 2.43 (q, *J=*7.6 Hz, 2 H, C*H*_2_CH_3_), 1.83 (s, 3 H, C*H*_3_), 1.05 (t, *J=*7.6 Hz, 3 H, CH_2_C*H*_3)_  ppm; ^**13**^ **C{^1^H} NMR** (101 MHz, [D_6_]DMSO, 297 K): *δ*=171.3, 167.8, 149.3, 146.5, 130.5, 94.4, 16.9, 14.0, 8.1 ppm; **IR (ATR)**: ν=3409 (m; O−H), 3287 (m; N−H), 3173–2187 (m, br; O−H⋅⋅⋅O), 1725 (m; C=O), 1660 (s; C=O, Amide I), 1633 (s; C=C) cm^−1^; **MS (DEI)**: *m*/*z* (%)=181 (54) [M]^+^, 164 (13), 137 (100), 122 (30), 108 (18); **Elemental analysis** (%): calcd for C_9_H_11_NO_3_ (181.19): C 59.66, H 6.12, N 7.73; found: C 59.68, H 6.13, N 7.79.

*(Z)‐2‐(3‐ethyl‐4‐methyl‐5‐oxo‐1,5‐dihydro‐2H‐pyrrol‐2‐ylidene)ethanoyl chloride (**4**)*: To a solution of **3** (250 mg, 1.38 mmol) in anhydrous DCM (8.5 mL) were added oxalyl chloride (227 mg, 1.79 mmol) and anhydrous DMF (one drop). The mixture was stirred for 2 h at room temperature in a nitrogen atmosphere. The solvent was removed by vacuum distillation and the residue dried in vacuo, providing **4** as a pale brown solid, which was used for the next step without further purification (271 mg, 98 %).

^**1**^ **H NMR** (400 MHz, [D_8_]THF, 297 K): *δ*=9.79 (s, 1 H, N*H*), 5.72 (s, 1 H, C*H*), 2.48 (q, *J=*7.6 Hz, 2 H, C*H*_2_CH_3_), 1.89 (s, 3 H, C*H*_3_), 1.12 (t, *J=*7.6 Hz, 3 H, CH_2_C*H*_3_) ppm; ^**13**^ **C{^1^H} NMR** (101 MHz, [D_8_]THF, 297 K): *δ*=172.6, 164.5, 154.6, 147.5, 133.9, 97.6, 18.0, 14.1, 8.4 ppm; **MS (DEI)**: *m*/*z* (%)=199 (11) [M]^+^, 164 (100), 136 (19), 108 (10); **HRMS (EI)**: *m*/*z* calcd for C_9_H_10_ClNO_2_: 199.0400; found: 199.0402 [M]^+^.

*(Z)‐2‐(3‐ethyl‐4‐methyl‐5‐oxo‐1,5‐dihydro‐2H‐pyrrol‐2‐ylidene)ethanamide (dihydro Z‐BOX A)*: A solution of **4** (257 mg, 1.29 mmol) in anhydrous THF (25 mL) was cooled to 0 °C. Gaseous ammonia was bubbled through the solution for 0.5 h at 0 °C and was maintained for additional 0.5 h while warming up to room temperature. The solvent was removed in vacuo and the residue washed with water (3×5 mL). Purification by recrystallization from methanol provided pale yellow crystals of dihydro *Z*‐BOX A (164 mg, 71 %).

^**1**^ **H NMR** (400 MHz, [D_6_]DMSO, 297 K): *δ*=9.66 (s, 1 H, N*H*), 7.64 (s, 1 H, N*H*_2_), 7.22 (s, 1 H, N*H*_2_), 5.58 (s, 1 H, C*H*), 2.39 (q, *J=*7.6 Hz, 2 H, C*H*_2_CH_3_), 1.82 (s, 3 H, C*H*_3_), 1.07 (t, *J=*7.6 Hz, 3 H, CH_2_C*H*_3_) ppm; ^**13**^ **C{^1^H} NMR** (101 MHz, [D_6_]DMSO, 297 K): *δ*=170.9, 168.1, 146.3, 145.9, 129.8, 96.9, 17.1, 14.0, 8.1 ppm; **IR (ATR)**: ν=3346 (m; NH_2_), 3153 (m; NH_2_), 1696 (m; C=O, Amide I), 1664 (s; C=O, Amide I), 1610 (s; NH_2_, Amide II) cm^−1^; **MS (DEI)**: *m*/*z* (%)=180 (100) [M]^+^, 163 (46), 148 (12), 137 (49), 122 (23), 108 (10); **Elemental analysis** (%): calcd for C_9_H_12_N_2_O_2_ (180.21): C 59.99, H 6.71, N 15.55; found: C 60.01, H 6.83, N 15.88.

CCDC 1945467for dihydro BOX A contain the supplementary crystallographic data for this paper. These data are provided free of charge by The Cambridge Crystallographic Data Centre.

### Collection of biological samples

**Plant and insect material**: *Ipomoea batatas* Lam. cultivar Tainong 57 was grown for three weeks under long day conditions (16 h light with 100 μmol m^−2^ s^−1^, 8 h dark) at 28 °C (day) and 25 °C (night) at 70 % relative humidity.

*Spodoptera littoralis* (Boisd., Lepidoptera, Noctuidae) larvae were provided by Bayer Cropscience Germany and reared on an artificial diet.^49^, ^50^ The larvae were separated and starved 24 h prior to each feeding assay.

**Feces and spit collection**: Feces were collected from five different sets of *Spodoptera littoralis* larvae. Each set comprised ten 4^th^ instar larvae which were feeding on two *Ipomea batatas* plants (3 weeks‐old). After five hours of feeding, the larvae were removed and the feces collected after different incubation times (0, 1, 3, 5, and 7 days). For the first measured time‐point (0), the feces were collected immediately after removal of the larvae. The remaining feces were subsequently placed in two different conditions: low (dry) and high humidity (wet). High humidity (see above in plant and insect material) mimics the natural conditions of the herbivory plant system. Low humidity incubation (25 °C, 30 % humidity, 18 hours light) presents the laboratory condition of the insect room. Another set of feces was collected from *Helicoverpa armigera* and *Chelymorpha alternans* reared on *I. batatas* plants. Spit was collected from 5^th^ instar *S. littoralis* larvae reared on *I. batatas* plants. The larvae were gently squeezed on the second segment of the dorsal part to regurgitate the spit. The spit was pooled from 100 larvae. The feces and spit were kept in −20 °C until sample preparation.

**Fungal infected leaves collection**: *Alternaria brassicicola* was grown for two weeks at 22 °C on potato dextrose agar medium as described in literature.^51^ Subsequently, spore suspensions were freshly prepared by adding 5 mL of sterile H_2_O to the fungal mycelium and gently scraping off the spores and hyphae with a following filtration step through a sterile nylon membrane (75 μm pore size). The spore density was determined using a hemocytometer and adjusted to 2×10^6^ spores mL^−1^ with sterile H_2_O containing 0.01 % Tween‐20. *A. brassicicola* was kindly provided by the Jena Microbial Resource Center.

For the co‐cultivation with *A. brassicicola*, single leaves (*n*=10–12) of 3 week‐old *I. batatas* plants were cut and washed in tap water to remove the milky latex juice exuding from the cut stem. The detached leaves were each placed on round filter paper soaked in 1 mL sterile H_2_O in a square petri dish and infected with 5 μL of *A. brassicicola* spore suspension (2×10^6^ spores mL^−1^) or sterile water containing 0.01 % Tween‐20 as control. The plates were then sealed with parafilm and placed in humid conditions (see above) for 48 h.

**Leaf collection upon herbivory**: Five *Spodoptera littoralis* larvae (4^th^ instar) were placed on 3 leaves of a single sweet potato plant (3 weeks‐old) and allowed to feed for 30 minutes. Afterwards the treated leaves from 5 plants in total (*n*=15) were harvested and immediately frozen in liquid nitrogen until further extraction.

**Sample preparation**: All the obtained samples were dried for 2 d with a freeze dryer and ground in liquid N_2_. Three times around 20 mg were extracted with an adapted Bligh and Dyer method: After the addition of 0.2 mL CHCl_3_ and 0.4 mL MeOH the mixture was shaken for 5 min, which was repeated after the addition of 0.2 mL CHCl_3_ and after 0.2 mL H_2_O. After centrifuging, both supernatants were combined and dried with an evacuated centrifuge. After adding 70 μL H_2_O, shaking and centrifuging, the supernatants were measured twice with UHPLC‐MS. To evaluate the influence of the drying process, spit was extracted without drying and compared to extracts of dried samples. By comparing both results with normalization to the dry mass, a mean value of 34 % of the degradation products were observed after drying, compared to direct measurements of the liquid samples. This indicates that some compounds either degrade further or cannot be eluted again after drying.

For external calibration, standards of PDP A1/A2/B1/B2, BOX A/B/C, dihydro BOX A and HA were measured three times at concentrations between 0.01 μm and 5 μm.

**Data analysis**: The mean peak area of two measurements was normalized on the weighted dry mass and mean values and standard deviation was calculated from the three biological replicates. For statistical analysis, one‐way‐Anova with Bonferroni post‐hoc test was used.

## Conflict of interest

The authors declare no conflict of interest.

## Supporting information

As a service to our authors and readers, this journal provides supporting information supplied by the authors. Such materials are peer reviewed and may be re‐organized for online delivery, but are not copy‐edited or typeset. Technical support issues arising from supporting information (other than missing files) should be addressed to the authors.

Supplementary
