# Supplementary material for: Pyrrolic and Dipyrrolic Chlorophyll Degradation Products in Plants and Herbivores
Source: Chemistry. 2020 Apr 28;26(28):6205–13. doi: 10.1002/chem.201905236 (PMC7318184; doi:10.1002/chem.201905236)
Supplement: Supplementary file 1 — Supplementary [file CHEM-26-6205-s001.pdf]

Chemistry–A European Journal

Supporting Information

Pyrrolic and Dipyrrolic Chlorophyll Degradation Products in Plants and Herbivores

Marcel Ritter,[a] Vincensius S. P. Oetama,[b] Daniel Schulze,[c] Katrin Muetzlaff,[a] Anja K. Meents,[b] Raphael A. Seidel,[a] Helmar Görls,[c] Matthias Westerhausen,[c] Wilhelm Boland,[b] and Georg Pohnert*[a, b]

Table of Contents

I. Spectroscopic data 1
II. X-Ray crystal structure determinations 7
III. Mass spectrometry 8
IV. Statistical determinations 14
V. References

I. Spectroscopic data

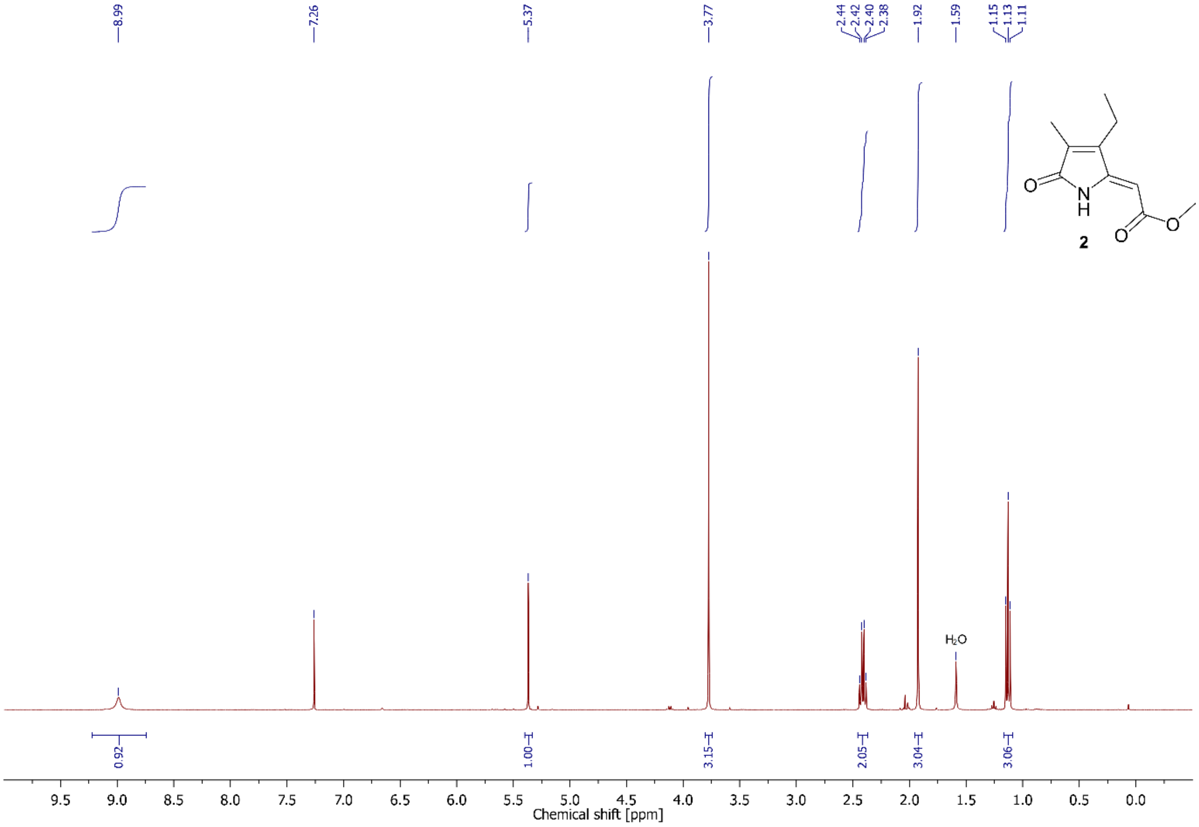

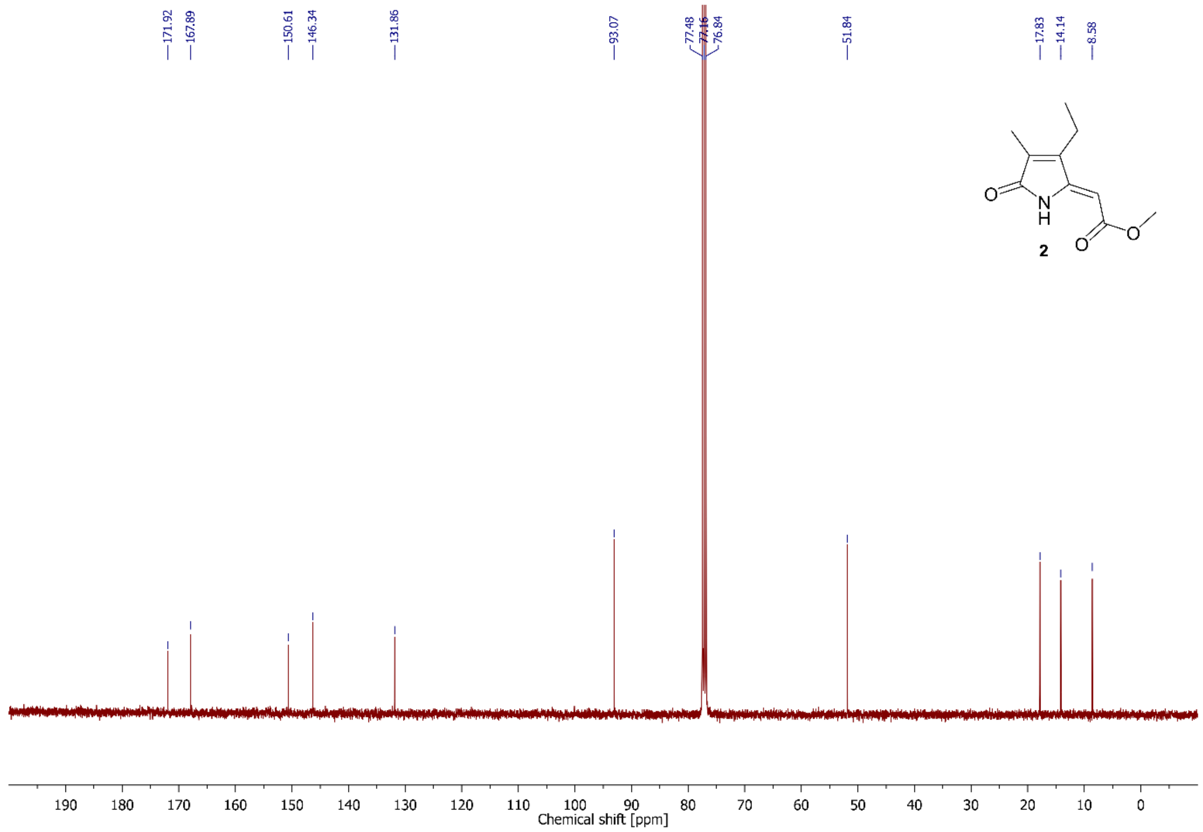

Figure S1: ^{1}H NMR spectrum (400 MHz, $CDCl_3$, 297 K, top) and ^{13}C{^{1}H} NMR spectrum (101 MHz, $CDCl_3$, 297 K, bottom) of **2**.

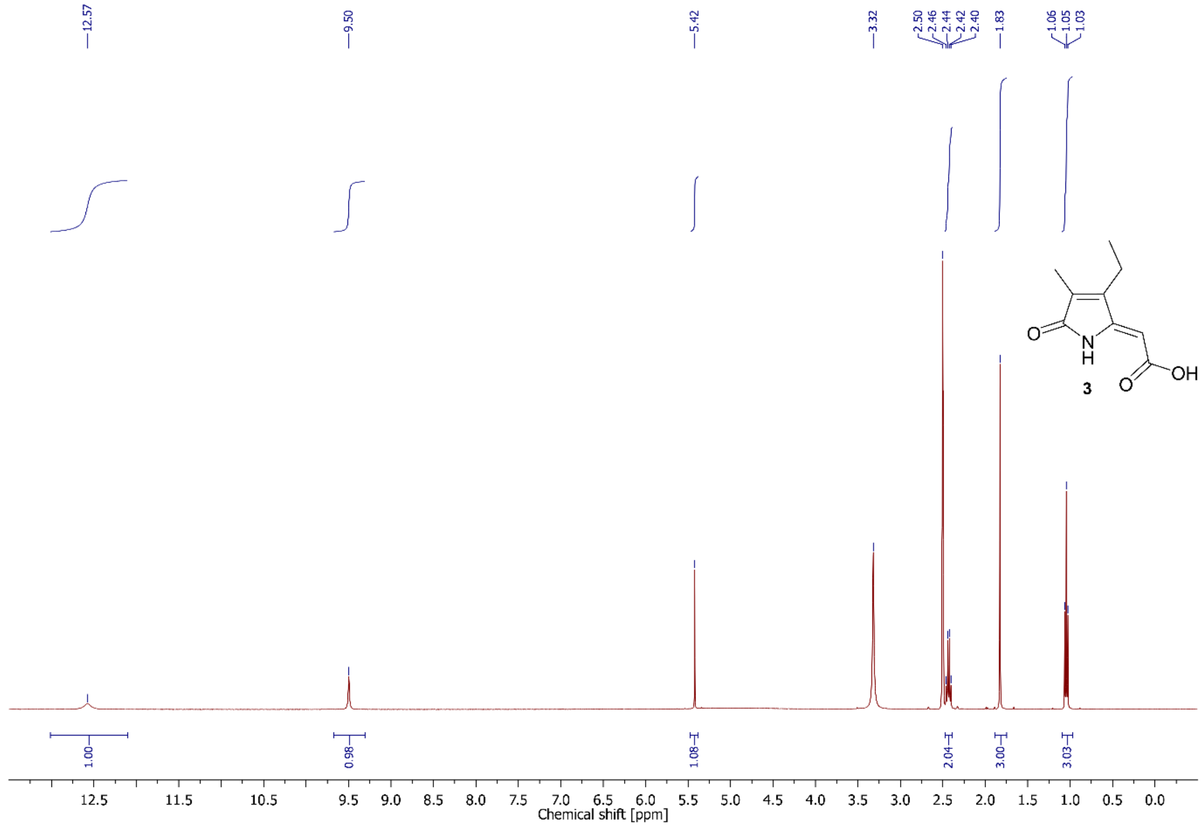

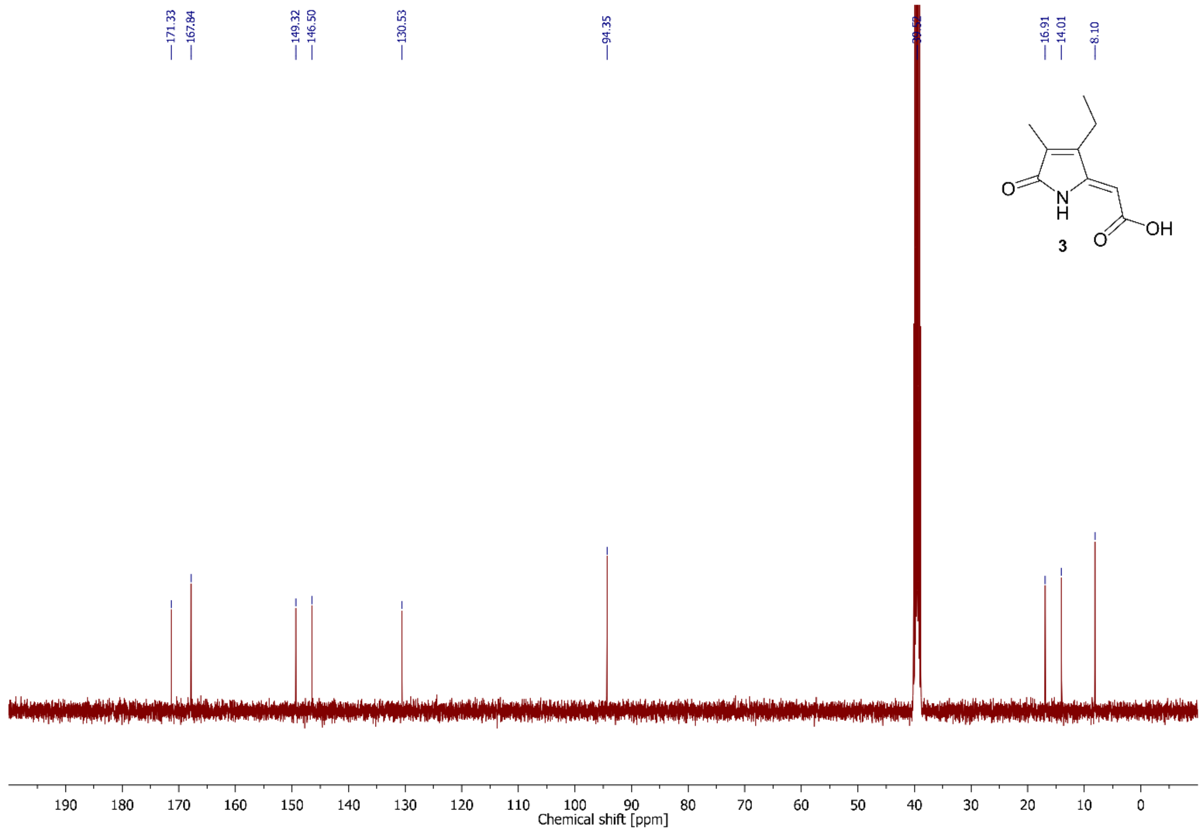

Figure S2: ^{1}H NMR spectrum (400 MHz, [D$_6$]DMSO, 297 K, top) and ^{13}C{^{1}H} NMR spectrum (101 MHz, [D$_6$]DMSO, 297 K, bottom) of **3**.

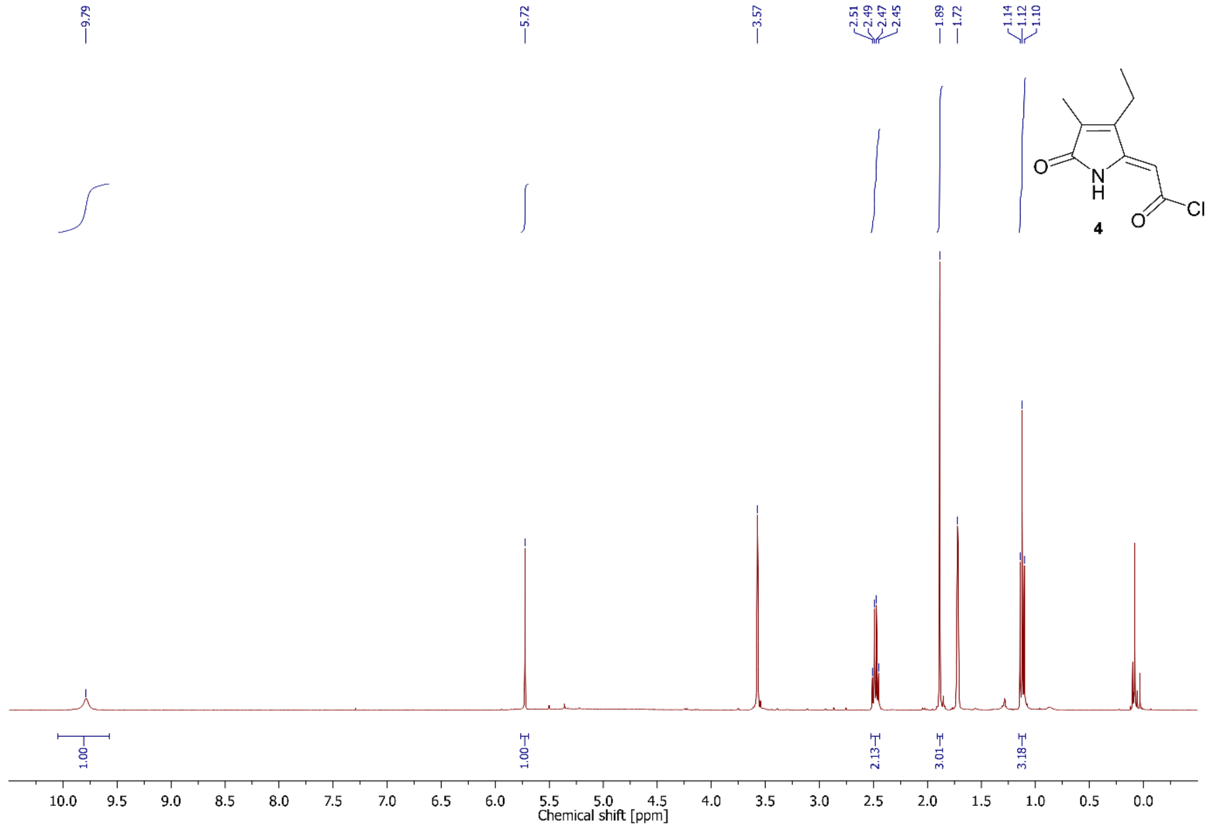

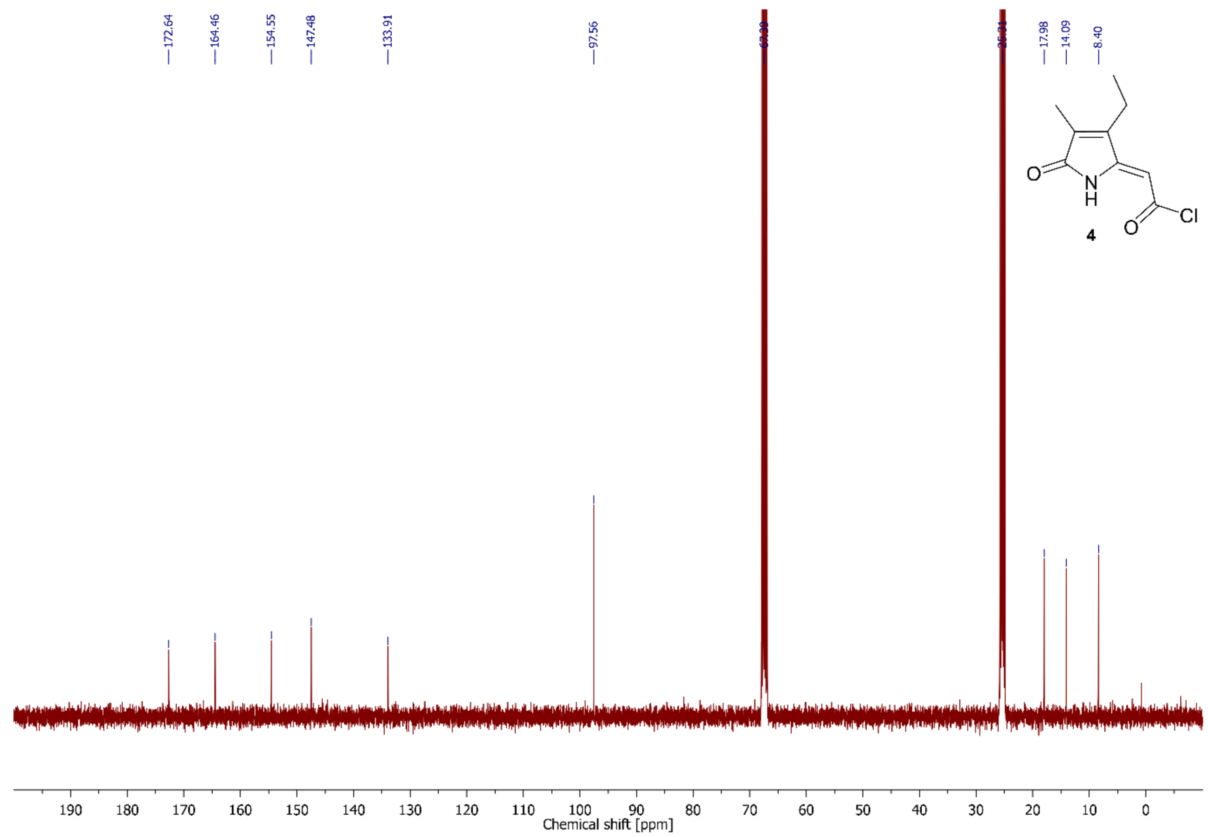

Figure S3: ^{1}H NMR spectrum (400 MHz, [D$_8$]THF, 297 K, top) and ^{13}C{^{1}H} NMR spectrum (101 MHz, [D$_8$]THF, 297 K, bottom) of **4**.

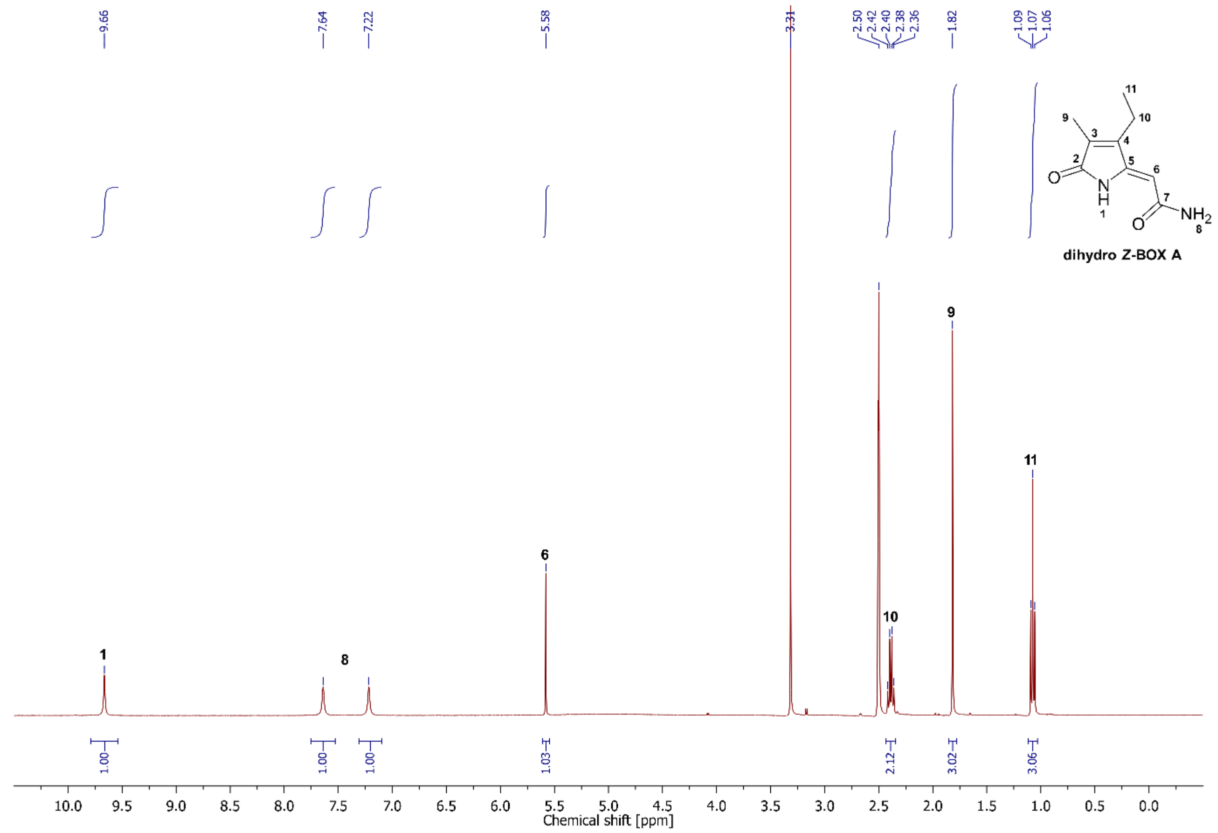

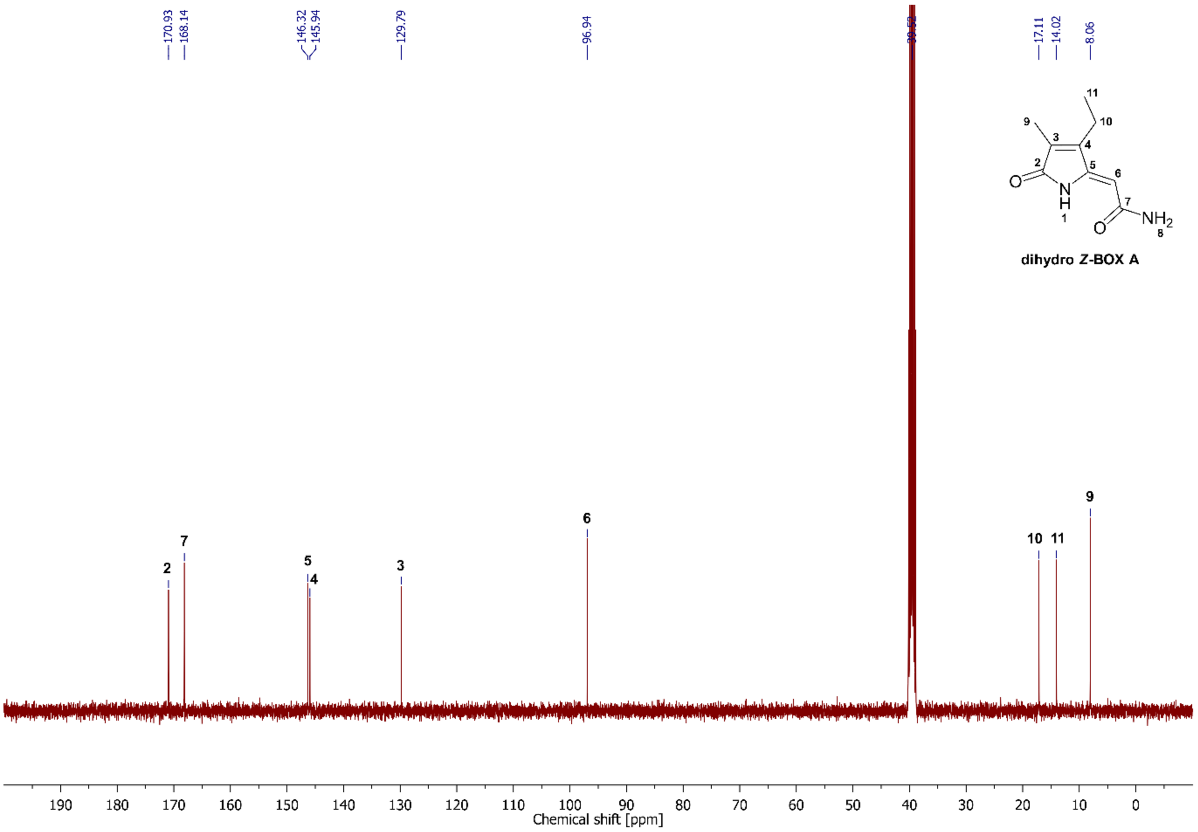

Figure S4: ^{1}H NMR spectrum (400 MHz, [D$_6$]DMSO, 297 K, top) and ^{13}C{^{1}H} NMR spectrum (101 MHz, [D$_6$]DMSO, 297 K, bottom) of **dihydro *Z*-BOX A**.

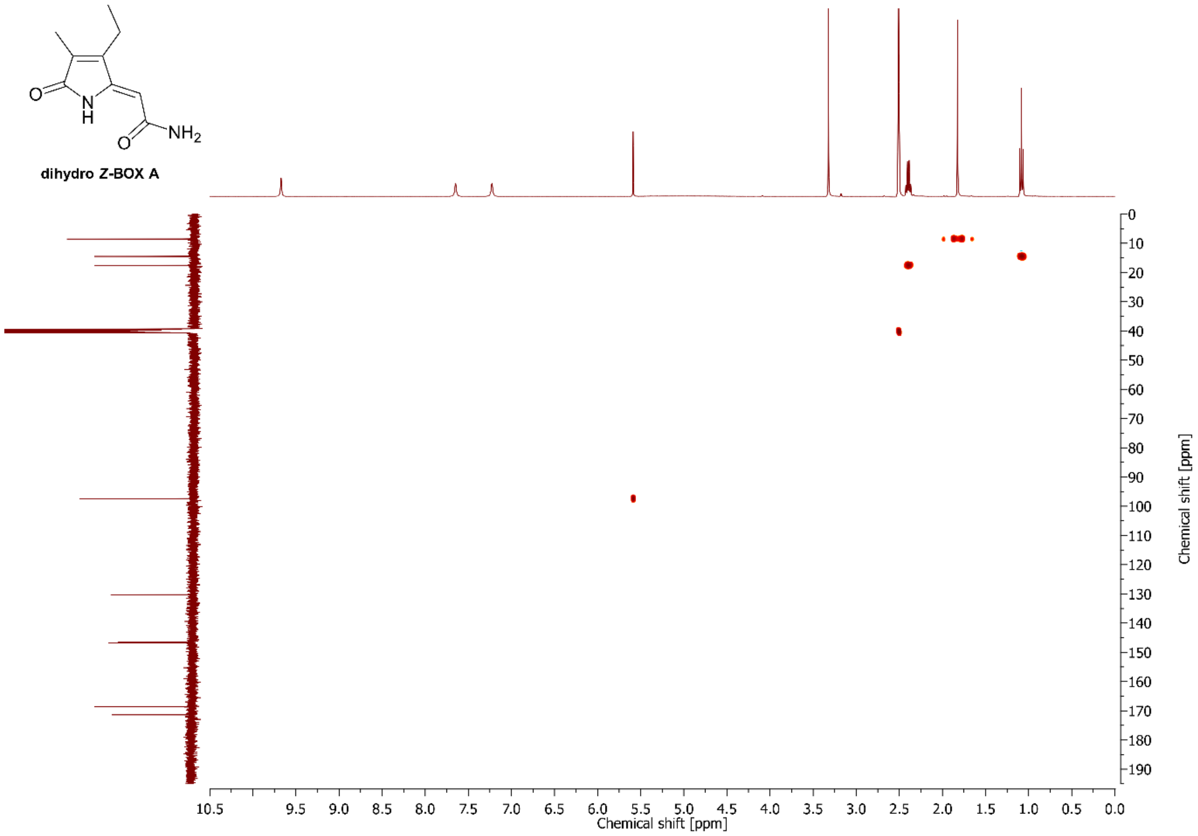

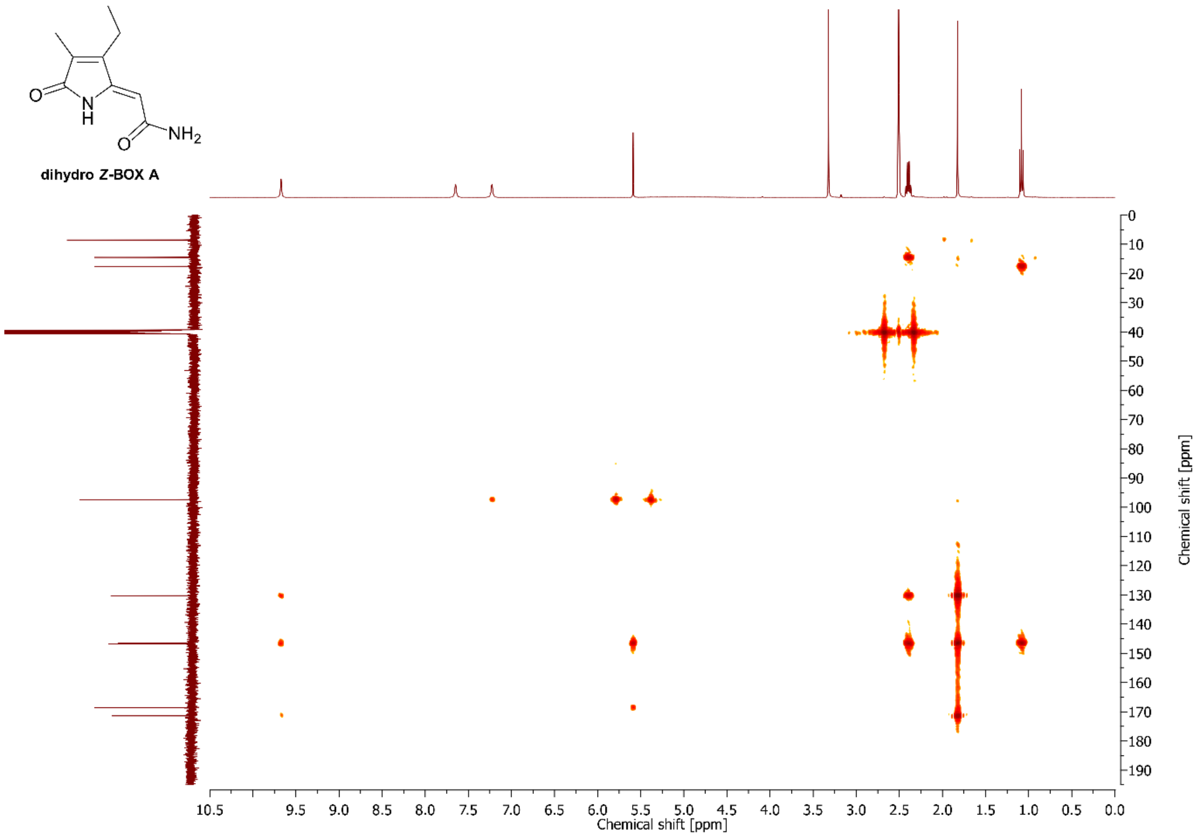

Figure S5: $^{1}H,^{13}C$-HSQC NMR spectrum (400 MHz, 101 MHz, [D$_6$]DMSO, top) and $^{1}H,^{13}C$-HMBC NMR spectrum (400 MHz, 101 MHz, [D$_6$]DMSO, bottom) of **dihydro *Z*-BOX A**.

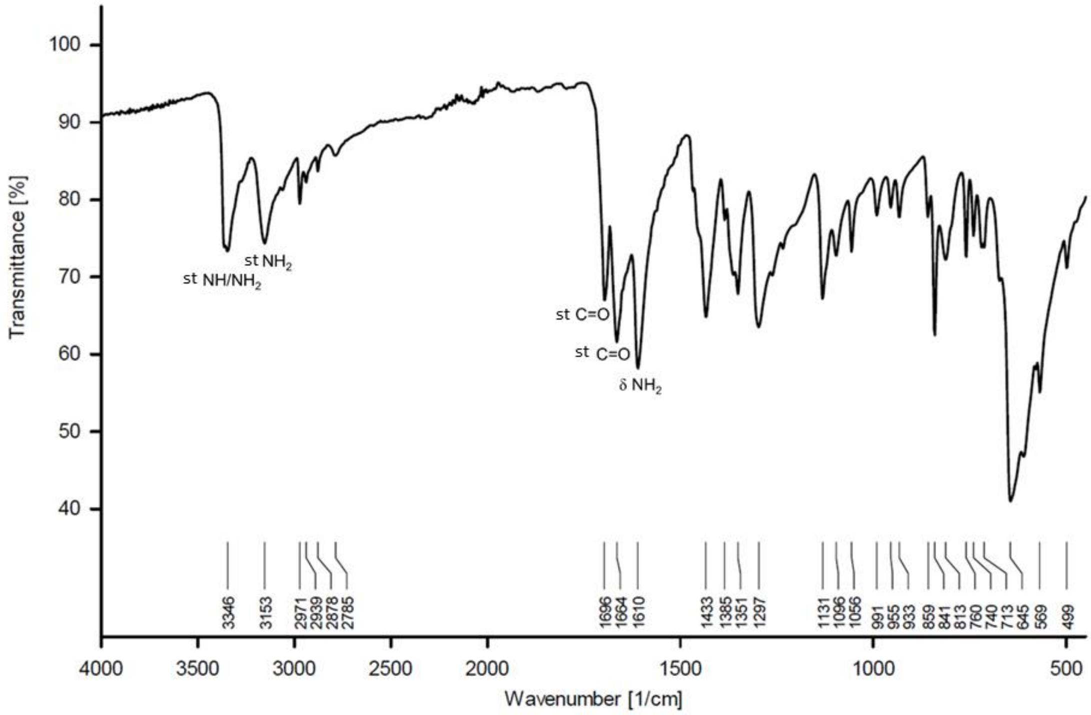

Figure S6: ATR-IR spectrum of **dihydro *Z*-BOX A**.

II. X-Ray crystal structure determinations

The intensity data were collected on a Nonius KappaCCD diffractometer, using graphite-monochromated Mo-K_{α} radiation. Data were corrected for Lorentz and polarization effects; absorption was taken into account on a semi-empirical basis using multiple-scans.[1-3]

The structure was solved by direct methods (SHELXS[4]) and refined by full-matrix least squares techniques against Fo^2 (SHELXL-97[4]). The hydrogen atoms bonded to the amine-groups N1 and N2 of **Dihydro-BOX A** were located by difference Fourier synthesis and refined isotropically. All other hydrogen atoms were included at calculated positions with fixed thermal parameters. All non-hydrogen atoms were refined anisotropically.[4]

XP (SIEMENS Analytical X-ray Instruments, Inc.) was used for structure representations.

Crystal Data for **Dihydro-BOX A**: $C_9H_{12}N_2O_2$, Mr = 180.21 $gmol^{-1}$, colourless prism, size 0.102 x 0.098 x 0.064 mm^3, triclinic, space group P $\bar{1}$, a = 7.2858(3), b = 7.8672(3), c = 8.7228(4) Å, α = 67.219(2), β = 80.643(3), γ = 76.091(2)°, V = 446.11(3) $Å^3$, T= -140 °C, Z = 2, $\rho_{calcd.}$ = 1.342 gcm^{-3}, μ (Mo-K_{α}) = .96 cm^{-1}, multi-scan, transmin: 0.6723, transmax: 0.7456, F(000) = 192, 6111 reflections in h(-8/9), k(-10/10), l(-8/11), measured in the range 2.86° ≤ Θ ≤ 27.47°, completeness Θ_{max} = 99.3%, 2034 independent reflections, R_{int} = 0.0395, 1635 reflections with $F_o > 4\sigma(F_o)$, 132 parameters, 0 restraints, $R1_{obs}$ = 0.0810, wR^2_{obs} = 0.2092, $R1_{all}$ = 0.1005, wR^2_{all} = 0.2284, GOOF = 1.106, largest difference peak and hole: 0.783 / -0.398 e $Å^{-3}$.

Supporting Information Available: Crystallographic data deposited at the Cambridge Crystallographic Data Centre under CCDC-1945467 for **Dihydro-BOX A** contain the supplementary crystallographic data excluding structure factors; this data can be obtained free of charge via www.ccdc.cam.ac.uk/conts/retrieving.html (or from the Cambridge Crystallographic Data Centre, 12, Union Road, Cambridge CB2 1EZ, UK; fax: (+44) 1223-336-033; or deposit@ccdc.cam.ac.uk).

[1] COLLECT, Data Collection Software; Nonius B.V., Netherlands, **1998**

[2] Z. Otwinowski & W. Minor, „Processing of X-Ray Diffraction Data Collected in Oscillation Mode“, in Methods in Enzymology, Vol. 276, Macromolecular Crystallography, Part A, edited by C.W. Carter & R.M. Sweet, pp. 307-326, Academic Press, San Diego, USA, **1997**

[3] SADABS 2016/2: Krause, L., Herbst-Irmer, R., Sheldrick G.M. & Stalke D., *J. Appl. Cryst.* 48 (2015) 3-10.

[4] Sheldrick, G. M. *Acta Cryst.* (2015). C**71**, 3-8.

III. Liquid Chromatography / Mass spectrometry

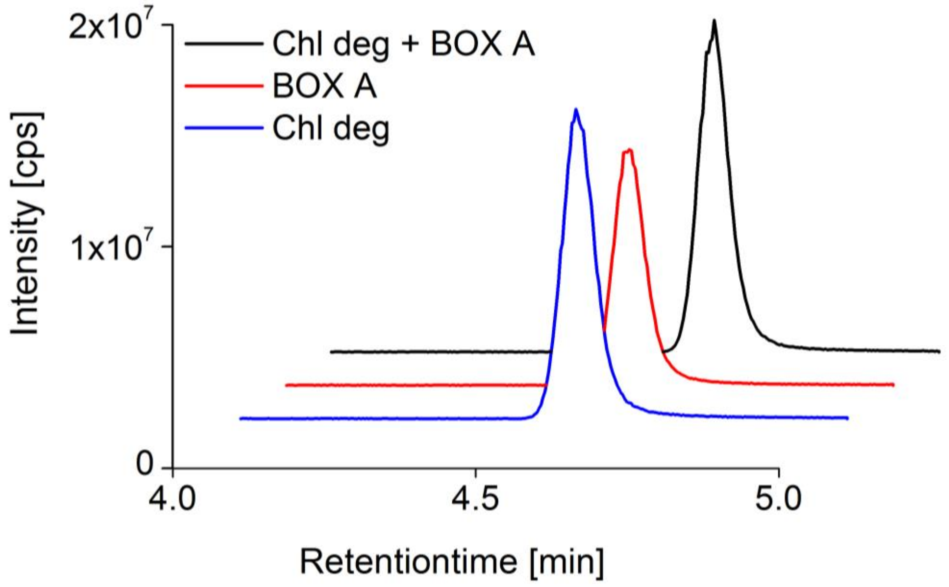

Figure S7: Chromatogram of the ion trace $[M+H]^+$ = 179.0816 monitoring Chl degradation products (blue), synthetic BOX A[1] (red) and injection of a mixture of isolated and synthetic BOX A (black).

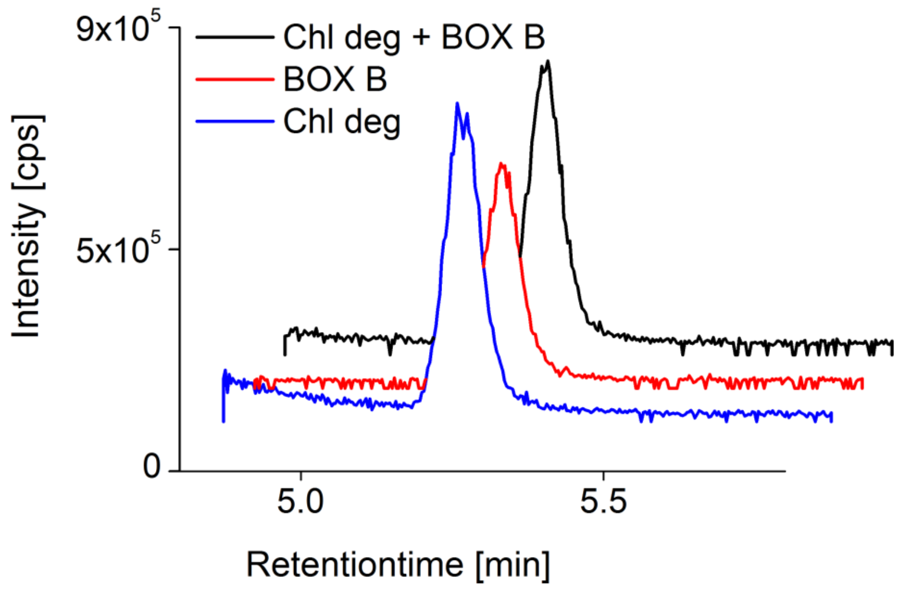

Figure S8: Chromatogram of the ion trace $[M+H]^+$ = 179.0816 monitoring Chl degradation products (blue), Synthetic BOX B[2] (red) and injection of a mixture of isolated and synthetic BOX A (black).

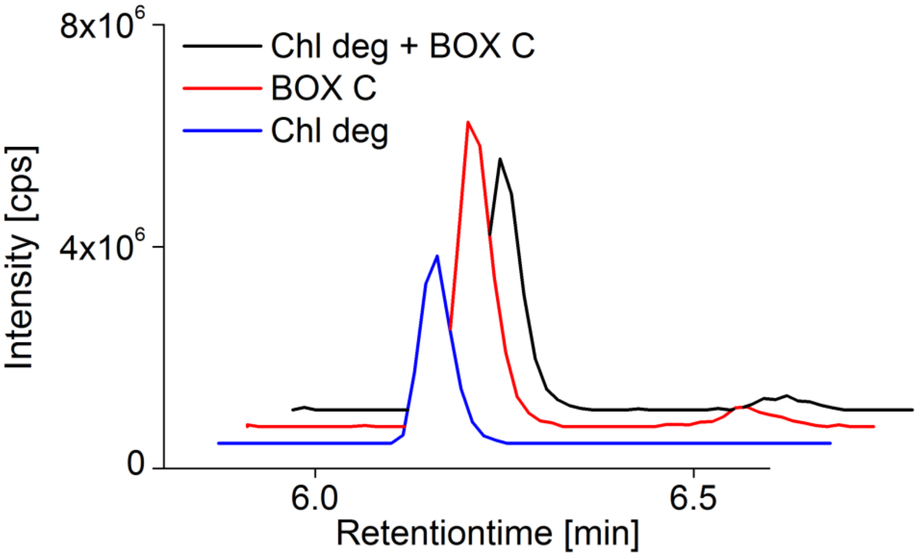

Figure S9: Chromatogram of the ion trace $[M+H]^+$ = 225,0871 monitoring Chl degradation products (blue), synthetic BOX C[3] (red) and injection of a mixture of isolated and synthetic BOX A (black).

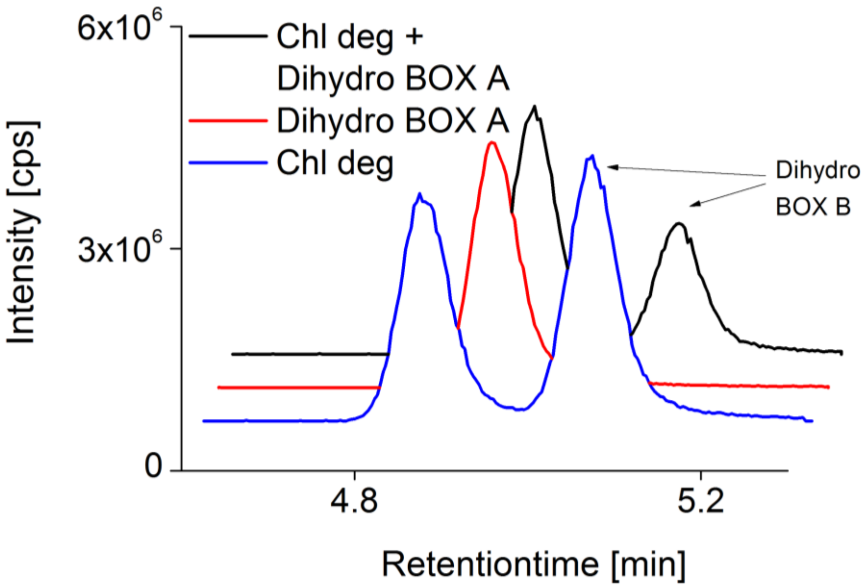

Figure S10: Chromatogram of the ion trace $[M+H]^+$ = 181.0973 monitoring Chl degradation products (blue), synthetic dihydro BOX A (red) and injection of a mixture of isolated and synthetic dihydro BOX A (black). The second signal can be tentatively assigned to dihydro BOX B given the retention time shift and the identical HR-MS spectrum (see Table S1).

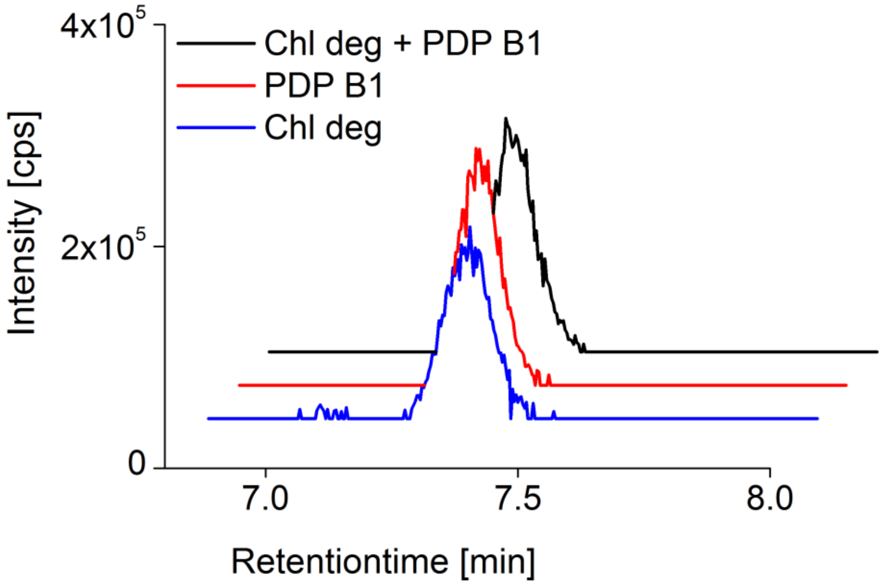

Figure S11: Chromatogram of the ion trace $[M+H]^+$ = 319.1290 monitoring Chl degradation products (blue), authentic PDP B1[4] (red) and injection of a mixture of isolated and authentic PDP B1 (black).

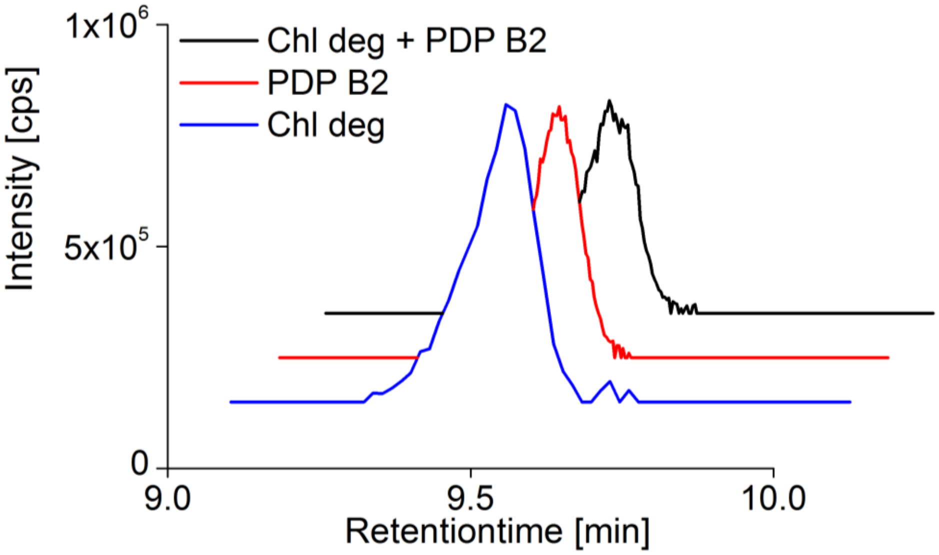

Figure S12: Chromatogram of the ion trace $[M+H]^+$ = 319.1290 monitoring Chl degradation products (blue), authentic PDP B2[4] (red) and injection of a mixture of isolated and authentic PDP B2 (black).

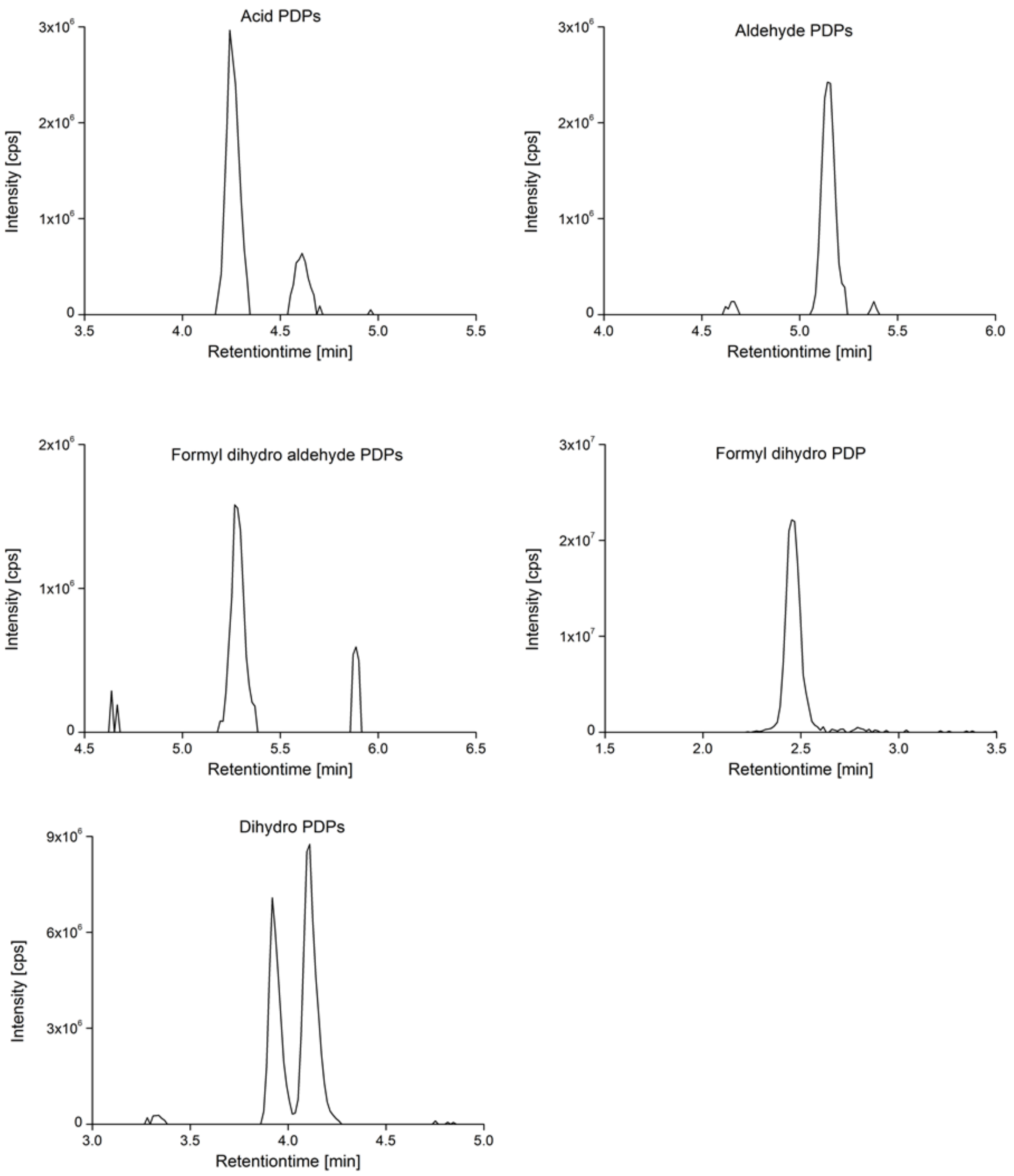

Figure S13: Chromatogram of the ion traces monitoring the putative novel PDPs depicted in Scheme 3. Acid PDPs $[M+H]^+$ = 347.1239; aldehyde PDPs $[M+H]^+$ = 331.1290; formyl dihydro aldehyde PDPs $[M+H]^+$ = 305.1133; formyl dihydro PDPs $[M+H]^+$ = 293.1130; dihydro PDPs $[M+H]^+$ =321.1444.

Table S1: Exact and accurate mass of the degradation products. In cases of isomeric compounds one entry is given for all isomers since Orbitrap HR-MS data are identical for the respective isomers.

Molecule	Sum formula $[M+H]^+$	Exact mass [u]	Accurate mass [u]	Δ [ppm]
PDPs	$C_{16}H_{19}O_5N_2$	319.1289	319.1290	0.48
BOX A/B	$C_9H_{11}O_2N_2$	179.0815	179.0816	0.65
BOX C	$C_{10}H_{13}O_4N_2$	225.0870	225.0871	0.43
Dihydro BOX A/B	$C_9H_{13}O_2N_2$	181.0973	181.0972	0.14
Hematinic acid	$C_8H_{10}O_4N$	184.0607	184.0604	0.24
Dihydro PDPs	$C_{16}H_{21}O_5N_2$	321.1445	321.14444	0.17
Formyl dihydro PDPs	$C_{14}H_{17}O_5N_2$	293.1132	293.11304	0.54
Formyl dihydro aldehyde PDPs	$C_{15}H_{17}O_5N_2$	305.1132	305.11328	0.27
Aldehyde PDPs	$C_{17}H_{19}O_5N_2$	331.1289	331.12906	0.64
Acid PDPs	$C_{17}H_{19}O_6N_2$	347.1238	347.12387	0.31

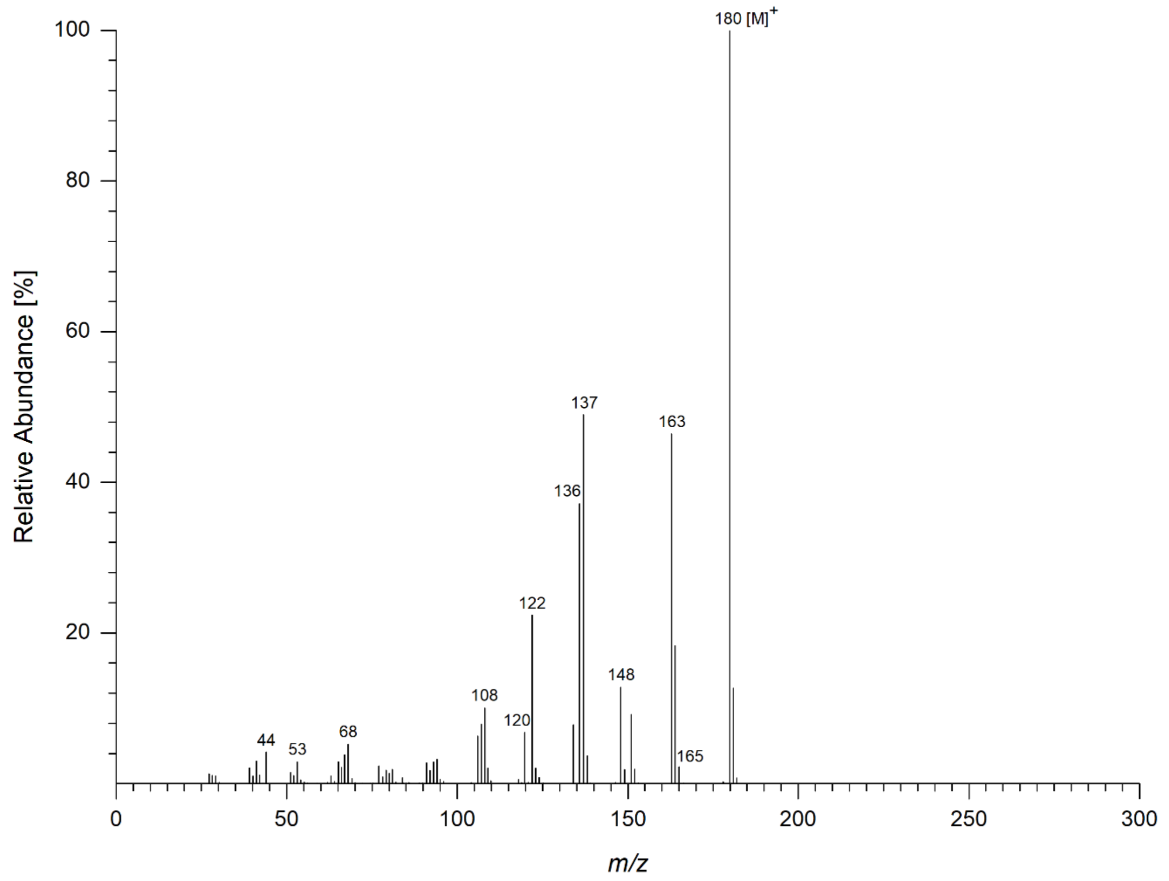

Figure S14: DEI-MS spectrum of **dihydro *Z*-BOX A**.

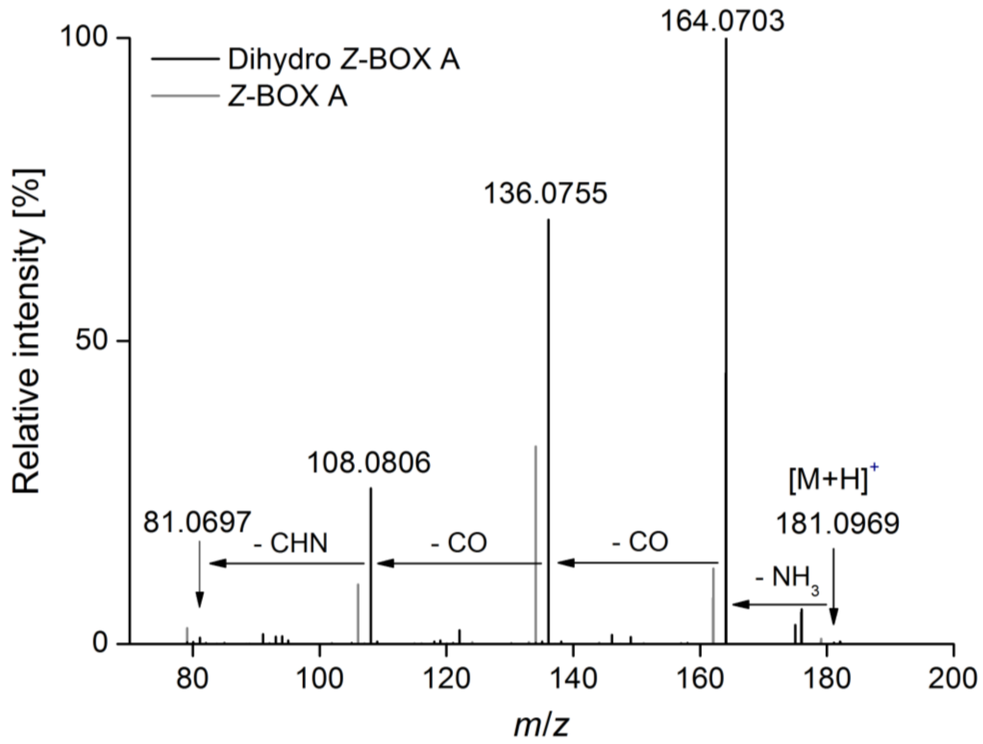

Figure S15: MS/MS spectra of **dihydro *Z*-BOX A** and *Z*-BOX A (grey).

IV. Statistical determinations

Table S2: Statistical comparison of the different samples with one-way-Anova and Bonferroni *post-hoc* test for Dihydro PDPs (*p* values are given).

**Dihydro PDPs**		Leaves	Leaves	Leaves	Leaves	Spit	Feces 0 d	Feces 7 d dry	Feces 7 d wet	Feces 0 d	Feces 0 d
			+ *S. littoralis*		+ *A. Brassicicola*		*S. littoralis*	*S. littoralis*	*S. littoralis*	*C. alternans*	*H. armigera*
Leaves			1	0.0137	0.3575	0.3833	0.5787	0.6640	0.4404	1	0.7184
Leaves	+ *S. littoralis*	1		1	1	0.0032	0.0049	0.0057	0.0037	1.0000	0.0062
Leaves		0.0137	1		1	2.2E-05	3.2E-05	3.7E-05	2.5E-05	0.0081	3.9E-05
Leaves	+ *A. Brassicicola*	0.3575	1	1		0.0004	0.0007	0.0008	0.0005	0.2149	0.0008
Spit	*S. littoralis*	0.3833	0.0032	2.2E-05	0.0004		1	1	1	0.6307	1
Feces 0 d	*S. littoralis*	0.5787	0.0049	3.2E-05	0.0007	1		1	1	0.9427	1
Feces 7 d dry	*S. littoralis*	0.6640	0.0057	3.7E-05	0.0008	1	1		1	1.0000	1
Feces 7 d wet	*S. littoralis*	0.4404	0.0037	2.5E-05	0.0005	1	1	1		0.7224	1
Feces 0 d	*C. alternans*	1	1.0000	0.0081	0.2149	0.6307	0.9427	1.0000	0.7224		1
Feces 0 d	*H. armigera*	0.71841	0.0062	3.9E-05	0.0008	1	1	1	1	1	

Table S3: Statistical comparison of the different samples with one-way-Anova and Bonferroni *post-hoc* test for Aldehyde PDPs (*p* values are given).

**Aldehyde PDPs**		Leaves	Leaves	Leaves	Leaves	Spit	Feces 0 d	Feces 7 d dry	Feces 7 d wet	Feces 0 d	Feces 0 d
			+ *S. littoralis*		+ *A. Brassicicola*		*S. littoralis*	*S. littoralis*	*S. littoralis*	*C. alternans*	*H. armigera*
Leaves			1	1	1	0.0182	0.0146	0.0043	0.0015	0.5293	0.0276
Leaves	+ *S. littoralis*	1		1	1	0.0406	0.0325	0.0096	0.0032	1	0.0615
Leaves		1	1		1	0.0621	0.0497	0.0147	0.0049	1	0.0938
Leaves	+ *A. Brassicicola*	1	1	1		0.1163	0.0932	0.0277	0.0092	1	0.1748
Spit	*S. littoralis*	0.0182	0.0406	0.0621	0.1163		1	1	1	1	1
Feces 0 d	*S. littoralis*	0.0146	0.0325	0.0497	0.0932	1		1	1	1	1
Feces 7 d dry	*S. littoralis*	0.0043	0.0096	0.0147	0.0277	1	1		1	1	1
Feces 7 d wet	*S. littoralis*	0.0015	0.0032	0.0049	0.0092	1	1	1		0.8529	1
Feces 0 d	*C. alternans*	0.5293	1	1	1	1	1	1	0.8529		1
Feces 0 d	*H. armigera*	0.0276	0.0615	0.0938	0.1748	1	1	1	1	1	

Table S4: Statistical comparison of the different samples with one-way-Anova and Bonferroni *post-hoc* test for Acid PDPs (*p* values are given).

**Acid PDPs**		Leaves	Leaves	Leaves	Leaves	Spit	Feces 0 d	Feces 7 d dry	Feces 7 d wet	Feces 0 d	Feces 0 d
			+ *S. littoralis*		+ *A. Brassicicola*		*S. littoralis*	*S. littoralis*	*S. littoralis*	*C. alternans*	*H. armigera*
Leaves			1	1	1	1	1	0.0160	0.8491	1	1
Leaves	+ *S. littoralis*	1		1	1	1	0.0587	0.8061	0.0057	1	0.0092
Leaves		1	1		1	1	1	1	0.3000	1	0.4795
Leaves	+ *A. Brassicicola*	1	1	1		1	1	0.3773	0.6538	1	1
Spit	*S. littoralis*	1	1	1	1		1	1	0.1365	1	0.2207
Feces 0 d	*S. littoralis*	1	0.0587	1	1	1		1	1	1	1
Feces 7 d dry	*S. littoralis*	0.0160	0.8061	1	0.3773	1	1		1	1	1
Feces 7 d wet	*S. littoralis*	0.8491	0.0057	0.3000	0.6538	0.1365	1	1		1	1
Feces 0 d	*C. alternans*	1	1	1	1	1	1	1	1		1
Feces 0 d	*H. armigera*	1	0.0092	0.4795	1	0.2207	1	1	1	1	

Table S5: Statistical comparison of the different samples with one-way-Anova and Bonferroni *post-hoc* test for Formyl dihydro aldehyde PDPs (*p* values are given).

**Formyl dihydro**		Leaves	Leaves	Leaves	Leaves	Spit	Feces 0 d	Feces 7 d dry	Feces 7 d wet	Feces 0 d	Feces 0 d
**aldehyde PDPs**			+ *S. littoralis*		+ *A. Brassicicola*		*S. littoralis*	*S. littoralis*	*S. littoralis*	*C. alternans*	*H. armigera*
Leaves			1	1	1	1.2E-05	1	1	1	1	1
Leaves	+ *S. littoralis*	1		1	1	2.1E-06	1	1	1	1	1
Leaves		1	1		1	2.3E-06	1	1	1	1	1
Leaves	+ *A. Brassicicola*	1	1	1		3.6E-06	1	1	1	1	1
Spit	*S. littoralis*	1.2E-05	2.1E-06	2.3E-06	3.6E-06		7.7E-05	1.4E-05	3.6E-06	1.4E-05	2.1E-06
Feces 0 d	*S. littoralis*	1	1	1	1	7.7E-05		1	1	1	1
Feces 7 d dry	*S. littoralis*	1	1	1	1	1.4E-05	1		1	1	1
Feces 7 d wet	*S. littoralis*	1	1	1	1	3.6E-06	1	1		1	1
Feces 0 d	*C. alternans*	1	1	1	1	1.4E-05	1	1	1		1
Feces 0 d	*H. armigera*	1	1	1	1	2.1E-06	1	1	1	1	

Table S6: Statistical comparison of the different samples with one-way-Anova and Bonferroni *post-hoc* test for Formyl dihydro PDPs (*p* values are given).

**Formyl dihydro PDPs**		Leaves	Leaves	Leaves	Leaves	Spit	Feces 0 d	Feces 7 d dry	Feces 7 d wet	Feces 0 d	Feces 0 d
			+ *S. littoralis*		+ *A. Brassicicola*		*S. littoralis*	*S. littoralis*	*S. littoralis*	*C. alternans*	*H. armigera*
Leaves			1	1	1	0.0001	0.3774	1	1	1	1
Leaves	+ *S. littoralis*	1		1	1	0.0001	0.5303	1	1	1	1
Leaves		1	1		1	0.0002	0.6319	1	1	1	1
Leaves	+ *A. Brassicicola*	1	1	1		0.0001	0.4794	1	1	1	1
Spit	*S. littoralis*	0.0001	0.0001	0.0002	0.0001		0.0765	0.0011	0.0020	0.0003	0.0003
Feces 0 d	*S. littoralis*	0.3774	0.5303	0.6319	0.4794	0.0765		1	1	1	0.9969
Feces 7 d dry	*S. littoralis*	1	1	1	1	0.0011	1		1	1	1
Feces 7 d wet	*S. littoralis*	1	1	1	1	0.0020	1	1		1	1
Feces 0 d	*C. alternans*	1	1	1	1	0.0003	1	1	1		1
Feces 0 d	*H. armigera*	1	1	1	1	0.0003	0.9969	1	1	1	

Table S7: Statistical comparison of the different samples with one-way-Anova and Bonferroni *post-hoc* test for BOX C (*p* values are given).

**BOX C**		Leaves	Leaves	Leaves	Leaves	Spit	Feces 0 d	Feces 7 d dry	Feces 7 d wet	Feces 0 d	Feces 0 d
			+ *S. littoralis*		+ *A. Brassicicola*		*S. littoralis*	*S. littoralis*	*S. littoralis*	*C. alternans*	*H. armigera*
Leaves			1	1	1	0.0051	1	1	1	0.2917	1
Leaves	+ *S. littoralis*	1		1	1	0.0053	1	1	1	0.3036	1
Leaves		1	1		1	0.0042	1	1	1	0.2394	1
Leaves	+ *A. Brassicicola*	1	1	1		0.0046	1	1	1	0.2667	1
Spit	*S. littoralis*	0.0051	0.0053	0.0042	0.0046		0.0025	0.0046	0.0034	1	0.0038
Feces 0 d	*S. littoralis*	1	1	1	1	0.0025		1	1	0.1418	1
Feces 7 d dry	*S. littoralis*	1	1	1	1	0.0046	1		1	0.2631	1
Feces 7 d wet	*S. littoralis*	1	1	1	1	0.0034	1	1		0.1949	1
Feces 0 d	*C. alternans*	0.2917	0.3036	0.2394	0.2667	1	0.1418	0.2631	0.1949		0.2166
Feces 0 d	*H. armigera*	1	1	1	1	0.0038	1	1	1	0.2166	

Table S8: Statistical comparison of the different samples with one-way-Anova and Bonferroni *post-hoc* test for PDPs (*p* values are given).

**PDPs**		Leaves	Leaves	Leaves	Leaves	Spit	Feces 0 d	Feces 7 d dry	Feces 7 d wet	Feces 0 d	Feces 0 d
			+ *S. littoralis*		+ *A. Brassicicola*		*S. littoralis*	*S. littoralis*	*S. littoralis*	*C. alternans*	*H. armigera*
Leaves			1	1	1	0.9587	1	1	1	0.0008	1
Leaves	+ *S. littoralis*	1		1	1	1	1	1	1	0.0022	1
Leaves		1	1		1	0.7989	1	1	1	0.0007	1
Leaves	+ *A. Brassicicola*	1	1	1		0.6237	1	1	1	0.0005	1
Spit	*S. littoralis*	0.9587	1	0.7989	0.6237		0.9882	1	1	0.2694	1
Feces 0 d	*S. littoralis*	1	1	1	1	0.9882		1	1	0.0009	1
Feces 7 d dry	*S. littoralis*	1	1	1	1	1	1		1	0.0013	1
Feces 7 d wet	*S. littoralis*	1	1	1	1	1	1	1		0.0018	1
Feces 0 d	*C. alternans*	0.0008	0.0022	0.0007	0.0005	0.2694	0.0009	0.0013	0.0018		0.0170
Feces 0 d	*H. armigera*	1	1	1	1	1	1	1	1	0.0170	

Table S9: Statistical comparison of the different samples with one-way-Anova and Bonferroni *post-hoc* test for hematinic acid (*p* values are given).

**Hematinic acid**		Leaves	Leaves	Leaves	Leaves	Spit	Feces 0 d	Feces 7 d dry	Feces 7 d wet	Feces 0 d	Feces 0 d
			+ *S. littoralis*		+ *A. Brassicicola*		*S. littoralis*	*S. littoralis*	*S. littoralis*	*C. alternans*	*H. armigera*
Leaves			1	1	1	1	1	1	1	9.8E-13	1
Leaves	+ *S. littoralis*	1		1	1	1	1	1	1	9.8E-13	1
Leaves		1	1		1	1	1	1	1	1.6E-12	1
Leaves	+ *A. Brassicicola*	1	1	1		1	1	1	1	7.2E-13	1
Spit	*S. littoralis*	1	1	1	1		1	1	1	6.8E-13	1
Feces 0 d	*S. littoralis*	1	1	1	1	1		1	1	5.1E-13	1
Feces 7 d dry	*S. littoralis*	1	1	1	1	1	1		1	1.2E-12	1
Feces 7 d wet	*S. littoralis*	1	1	1	1	1	1	1		2.2E-12	1
Feces 0 d	*C. alternans*	9.8E-13	9.8E-13	1.6E-12	7.2E-13	6.8E-13	5.1E-13	1.2E-12	2.2E-12		3.0E-12
Feces 0 d	*H. armigera*	1	1	1	1	1	1	1	1	3.0E-12	

Table S10: Statistical comparison of the different samples with one-way-Anova and Bonferroni *post-hoc* test for dihydro BOX A (*p* values are given).

**Dihydro BOX A**		Leaves	Leaves	Leaves	Leaves	Spit	Feces 0 d	Feces 7 d dry	Feces 7 d wet	Feces 0 d	Feces 0 d
			+ *S. littoralis*		+ *A. Brassicicola*		*S. littoralis*	*S. littoralis*	*S. littoralis*	*C. alternans*	*H. armigera*
Leaves			1	1	1	1	1	1	0.0672	1	1
Leaves	+ *S. littoralis*	1		1	1	1	1	1	0.0672	1	1
Leaves		1	1		1	1	1	1	0.0672	1	1
Leaves	+ *A. Brassicicola*	1	1	1		1	1	1	0.0672	1	1
Spit	*S. littoralis*	1	1	1	1		1	1	0.2461	1	1
Feces 0 d	*S. littoralis*	1	1	1	1	1			0.0672	1	1
Feces 7 d dry	*S. littoralis*	1	1	1	1	1	1			1	1
Feces 7 d wet	*S. littoralis*	0.0672	0.0672	0.0672	0.0672	0.2461	0.0672	0.0672		0.0672	0.0672
Feces 0 d	*C. alternans*	1	1	1	1	1	1	1	0.0672		1
Feces 0 d	*H. armigera*	1	1	1	1	1	1	1	0.0672	1	

V References

[1] M. Klopfleisch, R. A. Seidel, H. Görls, H. Richter, R. Beckert, W. Imhof, M. Reiher, G. Pohnert, M. Westerhausen, *Org. Lett.* **2013**, *15*, 4608–4611.

[2] R. A. Seidel, B. Schowtka, M. Klopfleisch, T. Kühl, A. Weiland, A. Koch, H. Görls, D. Imhof, G. Pohnert, M. Westerhausen, *Tetrahedron Lett.* **2014**, *55*, 6526–6529.

[3] D. Schulze, J. Traber, M. Ritter, H. Görls, G. Pohnert, M. Westerhausen *Org. Biomol. Chem*. **2019**, *17*, 6489-6496

[4] M. Ritter, R. A. Seidel, P. Bellstedt, B. Schneider, M. Bauer, H. Görls, G. Pohnert, *Org. Lett.* **2016**, *18*, 4432–4435.
